# Supplementary material for: Structural models of genome-wide covariance identify multiple common dimensions in autism
Source: Nat Commun. 2024 Feb 27;15:1770. doi: 10.1038/s41467-024-46128-8 (PMC10899248; doi:10.1038/s41467-024-46128-8)
Supplement: Supplementary file 1 — Supplementary Information [file 41467_2024_46128_MOESM1_ESM.pdf]

Supplementary Information for

Structural models of genome-wide covariance identify multiple common dimensions in autism

Lucía de Hoyos[1], Maria T Barendse[1,2], Fenja Schlag[1], Marjolein MJ van Donkelaar[1], Ellen Verhoef[1], Chin Yang Shapland[3,4], Alexander Klassmann[5], Jan Buitelaar[6-8], Brad Verhulst[9], Simon E Fisher[1,6], Dheeraj Rai[4,10,11], Beate St Pourcain[1,3,6] *

* Corresponding author. E-mail: beate.stpourcain@mpi.nl

INDEX

Supplementary Notes **3**

Supplementary Note 1. Phenotype subset identification and genetic proxy measures 3

Supplementary Note 2. Fitting structural models in GRM-SEM 5

Supplementary Note 3. GRM-SEM multi-factor multivariate simulations 9

Supplementary Methods **11**

Supplementary Methods 1. Genotype quality control in the SPARK cohort 11

Supplementary Methods 2. Genotype quality control in the SSC cohort 12

Supplementary Methods 3. Univariate polygenic scoring analysis in the SPARK Cohort 13

Supplementary Figures **14**

Supplementary Fig. 1 Individual and phenotype selection in the SPARK sample. 15

Supplementary Fig. 2 GREML heritability (h^2_{SNP}) estimates of phenotypes in SPARK. 16

Supplementary Fig. 3 GREML genetic correlations (r_g) across phenotypes in SPARK. 17

Supplementary Fig. 4 Proxy measure identification in SPARK. 18

Supplementary Fig. 5 Multi-factor GRM-SEM models in SPARK. 19

Supplementary Fig. 6 Phenotypic model in SPARK. 20

Supplementary Fig. 7 Individual and phenotype selection in the SSC sample. 21

Supplementary Fig. 8 Bi-factor model for the best-fitting model in SPARK. 22

Supplementary Fig. 9 Bi-factor model for the best-fitting model in SSC. 23

Supplementary Fig. 10 Comparison of h^2_{SNP} estimates between GREML and GRM-SEM. 24

Supplementary Fig. 11 Comparison of r_g estimates between GREML and GRM-SEM. 25

Supplementary Fig. 12 GRM-SEM multivariate model types. 26

Supplementary Fig. 13 Comparison of factor loadings estimated with EFA lavaan and GRM-SEM. 27

Supplementary Fig. 14 GRM-SEM simulations of a six-variate trait with two genetic factors without cross-loadings. 28

Supplementary Fig. 15 GRM-SEM simulations of a six-variate trait with two genetic factors with cross-loading. 29

Supplementary Fig. 16 GREML heritability (h^2_{SNP}) estimates of phenotypes in SSC. 30

Supplementary Fig. 17 Phenotypic correlations of phenotypes in SPARK. 31

Supplementary Fig. 18 Phenotypic correlations of phenotypes in SSC. 32

Supplementary Fig. 19 GREML h^2_{SNP} estimates of dichotomised ASD subcategories in SPARK. 33

Supplementary References **34**

Supplementary Notes

Supplementary Note 1. Phenotype subset identification and genetic proxy measures

To facilitate model convergence, we retained GREML genetic correlations across ASD phenotypes that passed the p-value threshold of $p(r_g) \leq 0.1$ (Supplementary Fig. 3). Based on the retained genetic correlations patterns, we selected subsets of phenotypes in SPARK for in-depth multivariate modelling (Supplementary Data 2).

Sequentially, we studied the genetic correlations of each of the 17 phenotypes with the other phenotypes and clustered phenotypes into related sets of measures. We observed four preliminary phenotype subsets implicating language/cognition-related phenotypes (Supplementary Data 2). These included two subsets related to (1) language disorder and (2) language level, as well as two subsets related to (3) cognitive age level and (4) language age level. Regarding behavioural problems and repetitive behaviour, we identified three preliminary phenotype subsets involving (5) oppositional defiant disorder (ODD), (6) Repetitive Behaviours Scale-Revised (RBSR) total score and (7) RBSR self-injurious scores. There was a single motor subset capturing genetic links with Developmental Coordination Disorder Questionnaire (DCDQ) proxies (8) as well as two preliminary phenotype subsets linked to developmental milestones, implicating the age of self feeding (9) and the age of crawling (10).

To avoid collinearity problems, highly correlated phenotypes measured with the same questionnaire were removed, retaining a single representative measure only, i.e. a proxy measure. To aid the selection of proxy measures, we first fitted one-factorial GRM-SEM IPC models (Supplementary Fig. 4) across scales within the same questionnaire to identify genetically similar measures (GRM-SEM r_g=1). Across developmental milestones (SPARK Background History Questionnaire, BGHX, Supplementary Fig. 4a-c), except for the age at which children started to self-feed with a spoon (age of self feeding), the selected BGHX scores (age of crawling, age of walking and age of sitting without support) genetically proxied

each other. Across motor phenotypes (DCDQ, Supplementary Fig. 4d-f), genetic variation was also shared and, thus, these scores (DCDQ total score, control during movement and fine motor handwriting) were considered genetic proxies. Similarly, for repetitive behaviour scales (RBSR, Supplementary Fig. 4g-i), except for a small specific genetic variance contribution to self-injurious behaviour, the studied RBSR scores (total score, ritualistic behaviour and sameness behaviour) proxied each other. Note that self-injurious behaviour was, consequently, not considered a proxy measure.

Eventually, we started the model-building process by selecting three phenotype subsets that (i) comprehensively represented all other preliminary subsets, (ii) captured largest the number of genetically interrelated measures and (iii) contained a single proxy measure only (Supplementary Fig. 3, Supplementary Data 2). The three phenotype subsets were related to developmental language disorder/delay (here forth referred to as language disorder, S_{DLD}), language level (S_{LL}) and age of crawling (S_{CRL}), respectively.

Supplementary Note 2. Fitting structural models in GRM-SEM

To model the multivariate genetic architecture as captured by the selected phenotype subsets (Supplementary Note 1) or combinations thereof, we carried out a series of genetic principal component analysis (PCA) eigenvalue decompositions, genetic exploratory factor analysis (EFA) steps and, finally, confirmatory factor analysis (GRM-SEM) steps (Fig. 1b, Methods), in both SPARK and SSC. Across all subsets, EFA oblimin predicted factor correlations were modest (see below) and EFA oblimin model produced a similar pattern of loadings as EFA varimax. GRM-SEM models were, thus, informed by EFA varimax predictions. Each of the presented models below fitted the data best, based on AIC and BIC, with a fit that was comparable to a saturated (Cholesky) model and a bi-factor model (Supplementary Data 3), where the latter confirmed the predicted independence of genetic factors.

For the S_{DLD} phenotype subset (Supplementary Fig. 5a-c), we investigated genomic covariance across three language/cognition phenotypes (language disorder, cognitive age level, language level), general behaviour (oppositional defiant disorder (ODD)), a developmental milestone (age of self feeding), a motor score proxy (DCDQ total score) and a repetitive behaviour proxy (RBSR total score). Eigenvalue decomposition of Cholesky-derived genetic trait correlations identified two genetic dimensions (Supplementary Fig. 5a) that were modestly genetically correlated (r=0.26), consistent with factorial independence. The best-fitting model, based on AIC and BIC, was an IPC model with two genetic factors (Supplementary Data 3), closely matching the fit of the saturated model (p_{LRT}>0.99) and the bi-factor model (p_{LRT}>0.99). We identified a common genetic factor accounting, predominantly, for variation in language performance (A_{lang}) with the strongest factor loading for language level (λ_{lang}=0.47,SE=0.08), and a factor explaining variation in behavioural problems (A_{beh}) with the strongest factor loading for liability to ODD (λ_{beh}=0.41,SE=0.09) (Supplementary Fig. 5b-c).

The S_{LL} phenotype subset (Supplementary Fig. 5d-f) comprised four language/cognition phenotypes (language disorder, cognitive age level, language age level, language level), two developmental milestones (age of crawling, age of self feeding) and the self-injurious repetitive behaviour sub-phenotype (RBSR self-injurious). Genetic PCA eigenvalue decomposition of Cholesky-derived genetic trait correlations identified two genetic dimensions (Supplementary Fig. 5d) with little correlation (r=-0.03). Fitting structural models, informed by genetic EFA, revealed that a two-factor IPC model fitted the data best (Supplementary Data 3), based on AIC and BIC, with a fit close to the saturated model (p_{LRT}>0.99) and the bi-factor model (p_{LRT}>0.99). The first genetic factor explained, predominantly, better language performance (A_{lang}), with the strongest factor loading for language level (λ_{lang} =0.44,SE=0.08), as observed for S_{DLD}. However, the second genetic factor accounted for variation in developmental motor delay (A_{dev}), not behavioural problems, with the strongest factor loading for the age of crawling (λ_{dev}=0.46,SE=0.12, Supplementary Fig. 5e-f).

For the third phenotype subset, age of crawling (S_{CRL}), genetic PCA eigenvalue decomposition did not reveal a clear factor dimension (Supplementary Fig. 5g). Therefore, we proceeded to combine phenotypes from the three subsets (S_{DLD},S_{LL},S_{CRL}) into a joint set (SPARK model, Fig. 3), facilitating the identification of a larger overarching genetic structure.

We retained phenotypes with the strongest factor loadings as well as cross-loadings from S_{DLD} and S_{LL} and combined them with all S_{CRL} phenotypes (Supplementary Data 2). Due to computational limitations, we excluded two S_{LL} phenotypes (cognitive age level and language age level) that were solely related to the language performance factor (Supplementary Fig. 5e-f) factor and highly correlated with the remaining measures (cognitive age level, language level: GREML r_g=0.87,SE=0.29; language age level, language level: GREML r_g=1.00,SE=0.24, Supplementary Fig. 3), showing similar association patterns in structural models.

The final SPARK model (Fig. 3, Supplementary Data 5) comprised two language phenotypes (language disorder, language level), general behaviour (ODD), two developmental milestones (age of crawling, age of self feeding), a DCDQ motor proxy (control during movement) and two RBSR repetitive behaviour scores (self-injurious behaviour, sameness behaviour). Genetic PCA eigenvalue decomposition of Cholesky-derived genetic trait correlations identified three genetic dimensions (Fig. 3a) with modest correlation (r=-0.34-0.06). Fitting structural models, informed by genetic EFA, revealed that a three-factor IPC model fitted the data best (Table 1), based on AIC and BIC, with a fit close to the saturated model (p_{LRT}>0.99). The first genetic factor explained, predominantly, better language performance (A_{lang}), with the strongest factor loading for language level (λ_{lang}=0.46,SE=0.08), as observed for S_{DLD} and S_{LL}. The second genetic factor accounted for variation in developmental motor delay (A_{dev}), with the strongest factor loading for the age of crawling (λ_{dev}=0.47,SE=0.10), as observed for S_{LL}. The third genetic factor accounted for variation in behavioural problems (A_{beh}), with the strongest factor loading for liability to ODD (λ_{beh}=0.45,SE=0.09), as observed for S_{DLD}.

The final SSC model (Fig. 4, Supplementary Data 7) comprised three language phenotypes (language disorder, language age level, language level), general behaviour (ODD), three developmental milestones (age of crawling, age of self feeding, age of walking), and the RBSR repetitive behaviour score (sameness behaviour). Eigenvalue decomposition of Cholesky-derived genetic trait correlations identified three genetic dimensions (Fig. 4a). Fitting structural models, informed by genetic EFA, revealed that a three-factor IPC model fitted the data best (Table 1), based on AIC and BIC, with a fit close to the saturated model (p_{LRT}>0.99). The first genetic factor (A_{F1}) explained, predominantly, language phenotypes, with the strongest factor loadings for the age of self feeding (λ_{F1} =-0.46,SE=0.19), as observed for the A_{lang} factor in the final SPARK model (Fig. 3). The second genetic factor (A_{F2}) accounted for variation in developmental motor delay, with the strongest factor loading for age of walking (λ_{F2}=0.62,SE=0.14), as observed for the A_{dev} factor in the final SPARK model (Fig. 3). The

third genetic factor (A_{F3}) accounted for variation that is shared across language, but also repetitive (RBSR sameness) behaviour, with the strongest factor loading for language age level (λ_{F3}=0.61,SE=0.10).

Supplementary Note 3. GRM-SEM multi-factor multivariate simulations

To confirm the robustness of the proposed data-driven genomic covariance modelling approach (Fig. 1b), we conducted simulations from a parametric model assessing evidence for bias. Path diagrams depicting a multi-factorial six-variate trait consisting of two genetic factors without (scenario 1) and with (scenario 2) cross-loadings are shown in Supplementary Fig. 14a and Supplementary Fig. 15a, respectively. The true values for factor loadings and corresponding genetic and residual variances are given in Supplementary Data 10-13. To reduce the computational burden, we assumed 2,000 individuals per trait and 5,000 causal loci conducting 20 simulations per scenario.

Besides the median estimate ($\hat{\beta}$) representing either factor loadings ($\hat{\lambda}$) or derived variances ($\hat{\theta}$), simulation performance measures were median bias, median empirical standard error (empSE) and coverage of 95%-confidence intervals (such that the estimated 95%-confidence interval contains the true value β) and the respective Monte-Carlo SEs (MCSE), as defined below (1):

$$bias = \frac{1}{N_{sim}}\sum_{i=1}^{N_{sim}}(\hat{\beta}_i - \beta\,), \qquad MCSE\ bias = \sqrt{\frac{1}{(N_{sim}-1)N_{sim}}\sum_{i=1}^{N_{sim}}\left(\hat{\beta}_i - \bar{\beta}\right)^2} \qquad (1)$$

$$EmpSE = \sqrt{\frac{1}{(N_{sim}-1)}\sum_{i=1}^{N_{sim}}\left(\hat{\beta}_i - \bar{\beta}\right)^2}, \qquad MCSE\ EmpSE = \frac{\widehat{EmpSE}}{\sqrt{2(N_{sim}-1)}} \qquad (2)$$

$$Coverage = \frac{1}{N_{sim}}\sum_{i=1}^{N_{sim}} 1(\hat{\beta}_{l,i} \leq \beta \leq \hat{\beta}_{u,i}\,), \quad MCSE\ Coverage = \sqrt{\frac{\widehat{Coverage}(1-\widehat{Coverage})}{N_{sim}}} \qquad (3)$$

Simulations predicted two underlying genetic factors from Cholesky-derived genetic trait correlations, throughout. IPC starting values and constraints for the genetic part were obtained from two-factor EFA varimax *lavaan* models fitted to a Cholesky-estimated genetic variance/covariance matrix (with diagonal inverse variance weights based on standard errors), given that EFA-oblimin-predicted correlations between genetic factors were near zero

(scenario 1: median EFA-predicted correlation: -0.020; scenario 2: median EFA-predicted correlation: $<-10^{-10}$). Subsequent GRM-SEM models were, thus, exclusively informed by EFA varimax factor predictions. EFA-derived genetic factor loadings <0.1 were constrained to zero. Among the 18 possible genetic factor loadings (based on a two-factor IPC model), the multi-step approach accurately identified zero and non-zero parameters with bias ranging between -0.035 to 0.007 for a six-variate trait without cross-loadings (scenario 1, Supplementary Data 10), and with bias between -0.030 to 0.015 for a six-variate trait with cross-loading (scenario 2, Supplementary Data 12). Consistent with the simulated trait architecture, on average, 33 genetic and residual factor loadings were estimated for scenario 1 (without cross-loading, Supplementary Data 10, median bias range: -0.035 to 0.022) and 34 factor loadings for scenario 2 (with cross-loading, Supplementary Data 12, median bias range: -0.030 to 0.030), confirming the robustness of the method. Coverage of factor loadings was sufficient and, taking MCSE into account, consistent with 95% or higher probability that confidence intervals contain the true value across estimated parameters, except one parameter in scenario 1 (94% probability). For derived genetic and residual trait covariance, based on the modelled six-variate traits in scenarios 1 and 2, median bias and coverage of true values were similar (Supplementary Data 11 and 13).

Supplementary Methods

Supplementary Methods 1. Genotype quality control in the SPARK cohort

Genetic data for the SPARK cohort were based on the SPARK 30K release (2) (Infinium Global Screening Array-24 v.1.0; N_{SNPs}=632,015; N_{ind}=27,064). Genotypes were lifted from Build38 to Build37 (hg19) and subjected to quality control (QC).

As part of individual QC measures, we excluded individuals due to sex mismatch, duplicated individuals, individual missingness (>3% missing data), non-European ancestry (MDS analysis; utilising all 1,000 Genomes Phase 3 populations as reference for clustering) and SPARK exclusion criteria. In line with SSC exclusion criteria, we excluded SPARK individuals with reported genetic diagnoses from the SPARK Basic Medical Measures questionnaire (e.g. Rett's Syndrome, DiGeorge syndrome, Fragile X) or due to serious environmental complications affecting central nervous system development (spina bifida, foetal alcohol syndrome, insufficient oxygen at birth, bleeding into the brain, serious prenatal infection, brain infection, lead poisoning or traumatic brain injury) or cognitive delays or impairment due to another medical condition or exposure (e.g. brain injury, stroke, lead poisoning, HIV, radiation, hydrocephalus, brain tumour, drug effects). The sample was eventually restricted to ASD probands only.

Variant QC excluded SNPs with a SNP missingness >5%, violations of Hardy-Weinberg equilibrium ($p<5x10^{-7}$), minor allele frequency (MAF) < 1% as well as non-autosomal SNPs.

After QC, a genetic relationship matrix (GRM) of 450,491 autosomal variants was created in PLINK applying a relationship cut-off of 0.05, based on a total of 5,331 individuals.

Supplementary Methods 2. Genotype quality control in the SSC cohort

Genetic data for the SSC cohort were based on the SSC Whole-genome 2 data release, including data from 3 arrays: Illumina Human1M v1.0 (N_{SNPs}=1,072,841; N_{ind}=1,354), Illumina Human1M-Duov3 (N_{SNPs}=1,199,033; N_{ind}=4,626), Illumina HumanOmni2.5 (N_{SNPs}=2,440,283; N_{ind}=4,240). Genotypes were lifted from Build 36 to Build 37 (hg19) and subjected to quality control (QC). For each array, individual and variant QC was carried out separately.

As part of individual QC measures, we excluded individuals due to sex mismatch, individual missingness (>3% missing data), non-European ancestry (MDS analysis; utilising all 1,000 Genomes Phase 3 populations as reference for clustering) and SSC exclusion criteria. The sample was eventually restricted to ASD probands only.

Variant QC excluded SNPs with a SNP missingness > 5% and violations of Hardy-Weinberg equilibrium (p<5x10^{-7}). Subsequently, genotype data were merged across the three arrays (N_{SNPs}= 2,757,032; N_{ind}= 1,966) and subjected to additional QC measures.

Additional QC measures on the merged file excluded individuals with >3% missing data and non-European ancestry (as described above). Furthermore, SNPs with >5% missingness, violations of HWE (p<5x10^{-7}), MAF<1% and non-autosomal SNPs were excluded.

After QC, a genetic relationship matrix (GRM) of 457,961 autosomal variants was created in PLINK applying a relationship cut-off of 0.05, based on a total of 1,946 individuals.

Supplementary Methods 3. Univariate polygenic scoring analysis in the SPARK Cohort

To compute PGS_{EA}, we used PRS-CS (3), a Bayesian-based approach that adjusts SNP effect sizes for linkage disequilibrium (LD) by applying a continuous-shrinkage parameter. The auto-option for a fully Bayesian estimation of the shrinkage parameter phi was selected. Furthermore, we used the software's default settings: 'a' in the gamma-gamma prior to 1, 'b' in the gamma-gamma prior to 0.5 and selecting 1,000 Markov Chain Monte Carlo iterations, 500 burn-in iterations, and a Markov chain thinning factor of 5. As LD reference file, we used the UK Biobank European reference panel recommended on the software's git-hub page (https://github.com/getian107/PRScs).

Summary statistics for educational attainment (4) were obtained from the SSGAC repository (https://thessgac.com/). The log odds of genetic SNP effects were aligned to indicate alleles with increased educational attainment. Once SNP effect sizes were calculated in PRS-CS, PGS_{EA} scores were calculated in PLINK (v1.9) (5) and, subsequently, Z-standardised.

PGS_{EA} were constructed for 5,331 unrelated SPARK individuals (genomic relatedness<0.05, see Supplementary Methods 1 for more details), based on high-quality imputed SNPs (INFO>0.8, 95%-posterior genotyping probability>0.9, MAF>0.005).

Supplementary Figures

Supplementary Fig. 1 Individual and phenotype selection in the Simons Foundation Powering Autism Research for Knowledge (SPARK) sample. 15

Supplementary Fig. 2 GREML heritability (h^2_{SNP}) estimates of phenotypes in SPARK........ 16

Supplementary Fig. 3 GREML genetic correlations (r_g) across phenotypes in SPARK. 17

Supplementary Fig. 4 Proxy measure identification in SPARK. 18

Supplementary Fig. 5 Multi-factor GRM-SEM models in SPARK. 19

Supplementary Fig. 6 Phenotypic model in SPARK. 20

Supplementary Fig. 7 Individual and phenotype selection in the Simons Simplex Collection (SSC) sample. 21

Supplementary Fig. 8 Bi-factor model for the best-fitting model in SPARK. 22

Supplementary Fig. 9 Bi-factor model for the best-fitting model in SSC. 23

Supplementary Fig. 10 Comparison of heritability estimates between GREML and GRM-SEM. 24

Supplementary Fig. 11 Comparison of genetic correlation estimates between GREML and GRM-SEM. 25

Supplementary Fig. 12 GRM-SEM multivariate model types**.** 26

Supplementary Fig. 13 Comparison of factor loadings estimated with EFA lavaan and GRM-SEM. 27

Supplementary Fig. 14 GRM-SEM simulations of a six-variate trait with two genetic factors without cross-loadings. 28

Supplementary Fig. 15 GRM-SEM simulations of a six-variate trait with two genetic factors with cross-loading. 29

Supplementary Fig. 16 GREML heritability (h^2_{SNP}) estimates of phenotypes in SSC. 30

Supplementary Fig. 17 Phenotypic correlations of phenotypes in SPARK. 31

Supplementary Fig. 18 Phenotypic correlations of phenotypes in SSC. 32

Supplementary Fig. 19 GREML heritability (h^2_{SNP}) estimates of dichotomised ASD subcategories in SPARK. 33

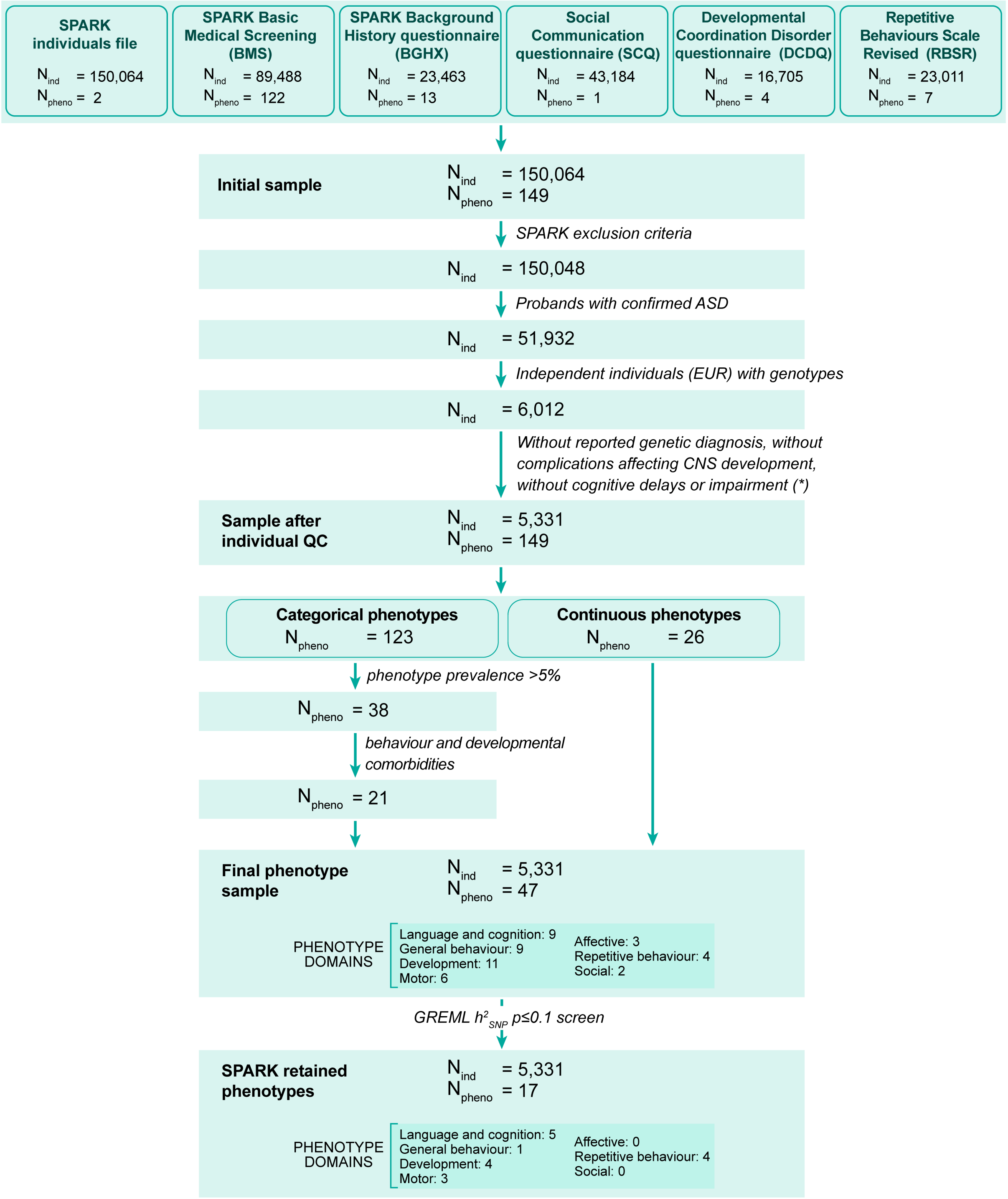

Supplementary Fig. 1 Individual and phenotype selection in the Simons Foundation Powering Autism Research for Knowledge (SPARK) sample. (*) See Supplementary Methods 1 for a more detailed description.

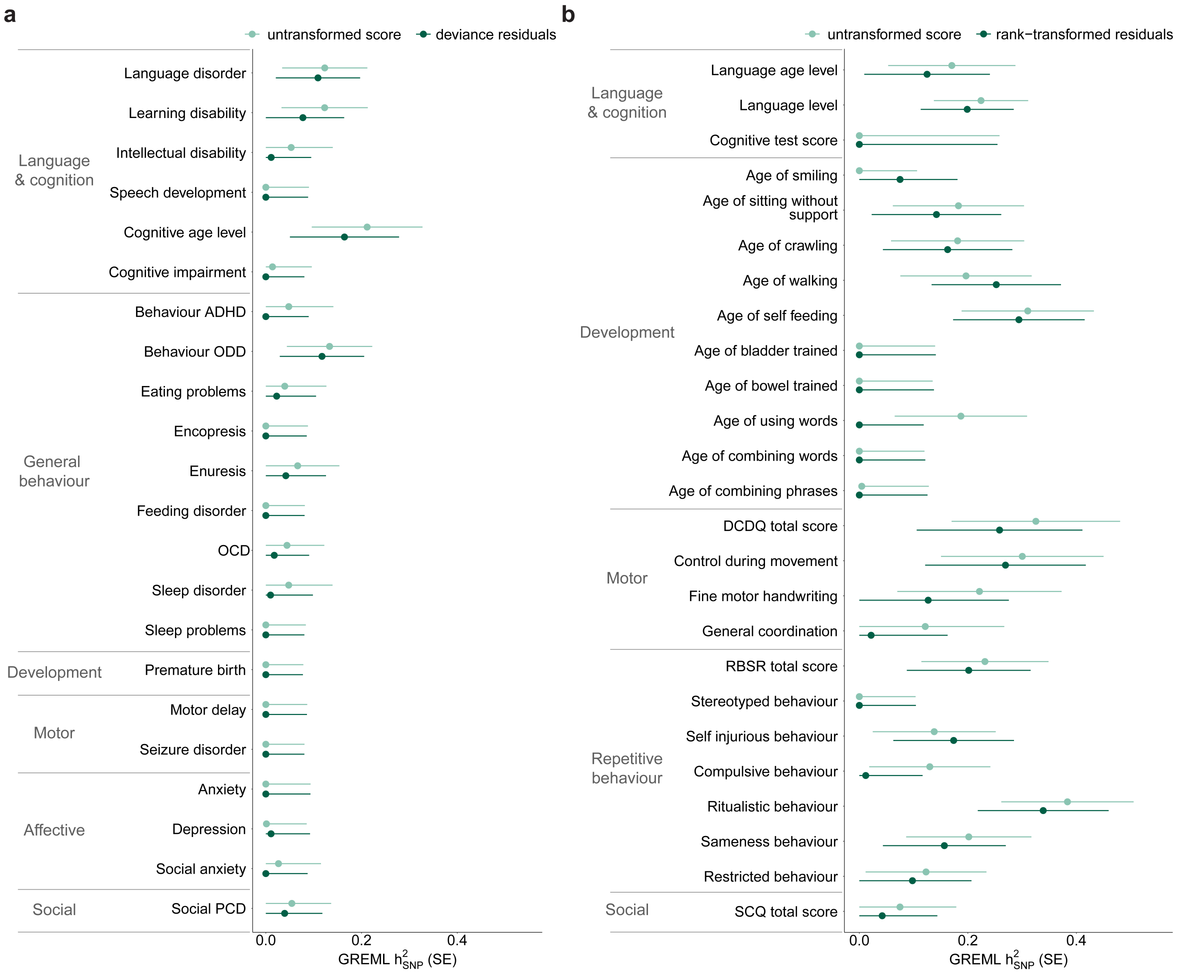

Supplementary Fig. 2 GREML heritability (h^2_{SNP}) estimates of phenotypes in SPARK. **a** GREML h^2_{SNP} estimates for categorical phenotypes are shown for untransformed scores (light green) and deviance residuals (dark green). **b** GREML h^2_{SNP} estimates for continuous phenotypes are shown for untransformed scores (light green) and rank-transformed residuals (dark green). **a**,**b** The error bars represent standard errors. GREML h^2_{SNP} estimates were adjusted for sex, age, age squared, and ten ancestry-informative principal components. For a detailed description of SPARK phenotypes see Supplementary Data 1. Abbreviations: ADHD (attention deficit hyperactivity disorder), DCDQ (Developmental Coordination Disorder Questionnaire), h^2_{SNP} (single nucleotide polymorphism-based heritability), OCD (obsessive-compulsive disorder), ODD (oppositional defiant disorder), PCD (pragmatic communication disorder), RBSR (Repetitive Behaviour Scale-Revised), SCQ (Social Communication Questionnaire).

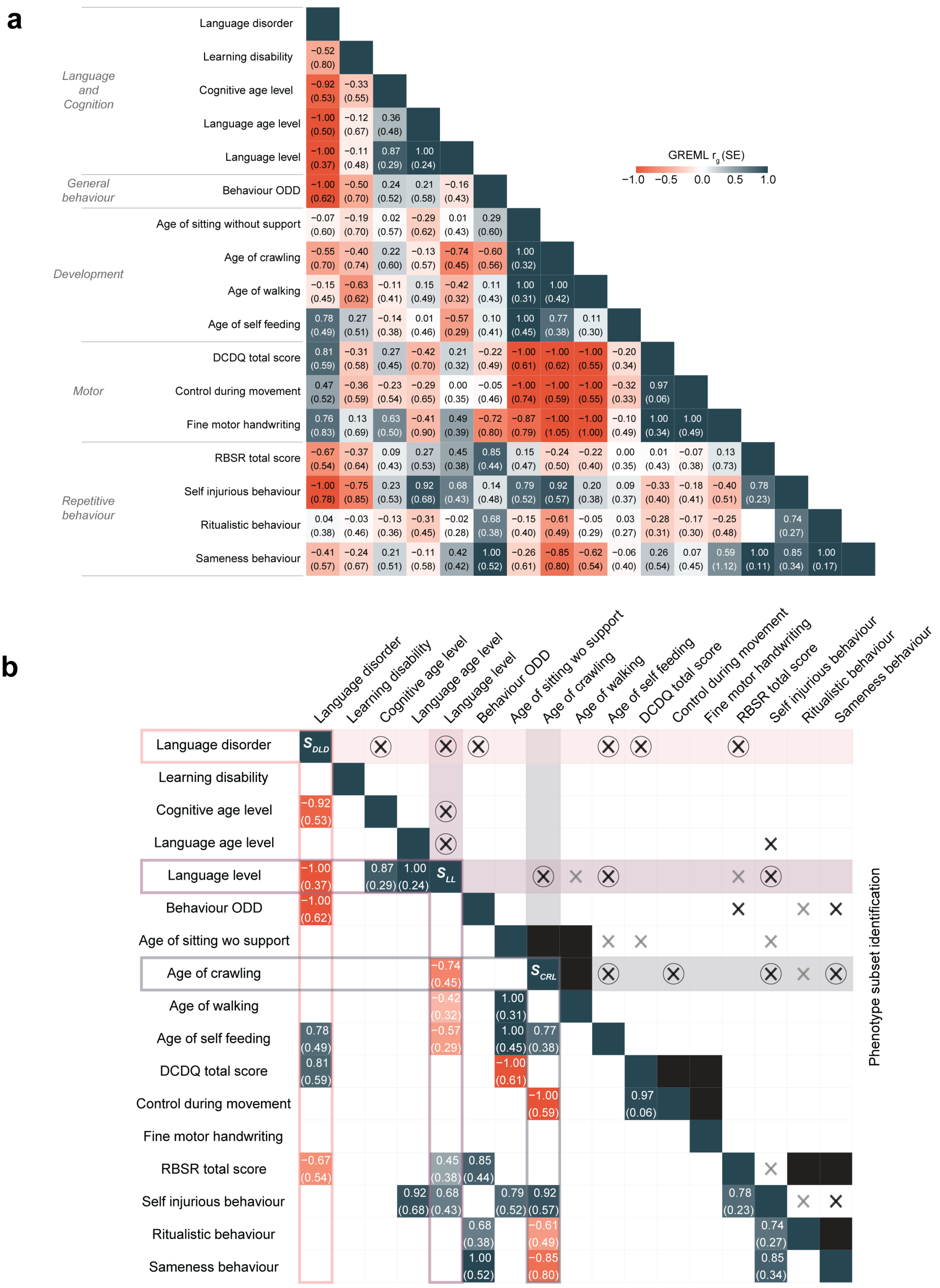

Supplementary Fig. 3 GREML genetic correlations (r_g) across phenotypes in SPARK. **a** Estimated GREML genetic correlations based on transformed scores (deviance residuals for categorical phenotypes and rank-transformed residuals for continuous, Npheno=17 with GREML h^2_{SNP} p≤0.1), adjusted for sex, age, age squared, and ten ancestry-informative principal components. **b** The lower triangle shows the genetic correlation screen (r_g) across ASD phenotypes as shown in panel **a**, passing $p(r_g)$≤0.1. The upper triangle shows the selected phenotype subsets that, together, comprehensively capture the genetic correlations (lower triangle) across studied phenotypes. Each phenotypic subset has a 'node' phenotype: S_{DLD} (language disorder), S_{LL} (language level) and S_{CRL} (age of crawling). Phenotypes within a subset are directly genetically correlated with the 'node' phenotype ($p(r_g)$≤0.1). The black boxes symbolise proxy phenotypes, as identified within uni-factorial GRM-SEM (rg=1, Supplementary Fig. 4). Circled 'x' within shaded boxes indicate the phenotypes that are included in each subset and were directly modelled with GRM-SEM. A black 'x' indicates directly estimated and a grey 'x' indirectly (proxied) genetic relationships. Phenotypes were adjusted for covariates and transformed into either rank-transformed residuals (continuous measures) or deviance residuals (categorical measures). Abbreviations: DCDQ (Developmental Coordination Disorder Questionnaire), ODD (oppositional defiant disorder), RBSR (Repetitive Behaviour Scale-Revised).

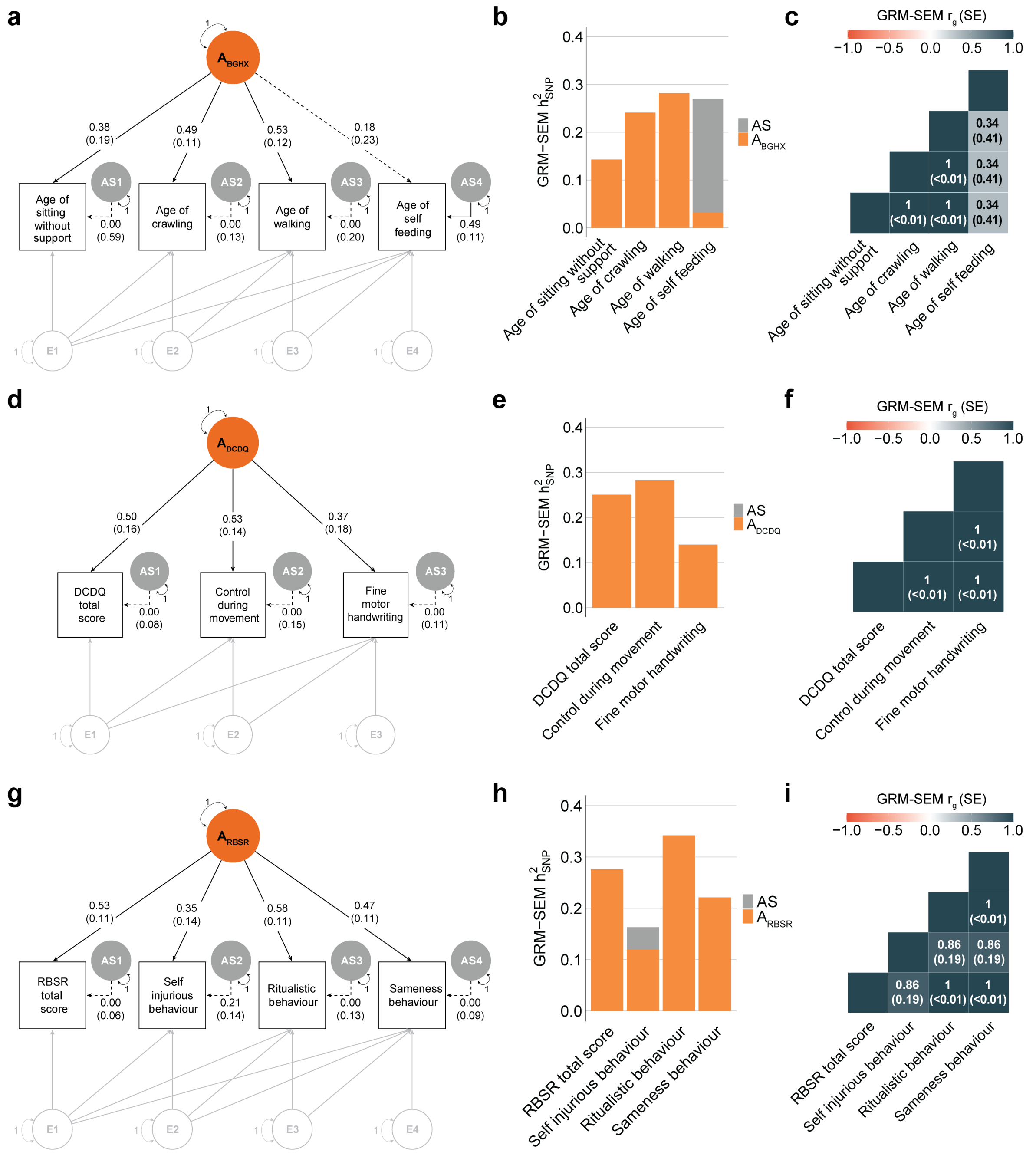

Supplementary Fig. 4 Proxy measure identification in SPARK. Hybrid GRM-SEM one-factor independent pathway/Cholesky (IPC) models were fitted across scores from the same questionnaire to identify shared genetic influences. **a** Path diagram, **b** standardised genetic variance plot and **c** genetic correlations for scores of the Background History Child Questionnaire (BGHX). **d** Path diagram, **e** standardised genetic variance plot and **f** genetic correlations for items across the Developmental Coordination Disorder Questionnaire (DCDQ). **g** Path diagram, **h** standardised genetic variance plot and **i** genetic correlations for items across the Repetitive Behaviour Scale-Revised (RBSR). **a,d,g** Observed measures are represented by squares and latent factors by circles. Single-headed arrows define factor loadings (shown with their corresponding SEs). The genetic part of the model has been modelled using an Independent Pathway model. Dotted and solid arrows define shared genetic factor loadings with p>0.05 and p≤0.05, respectively. The residual part has been modelled using a Cholesky model and all residual factor loadings are shown in grey. **c,f,i** SEs for GRM-SEM h^2_{SNP} contributions have been omitted for clarity. Abbreviations: h^2_{SNP} (single nucleotide polymorphism-based heritability), r_g (genetic correlation).

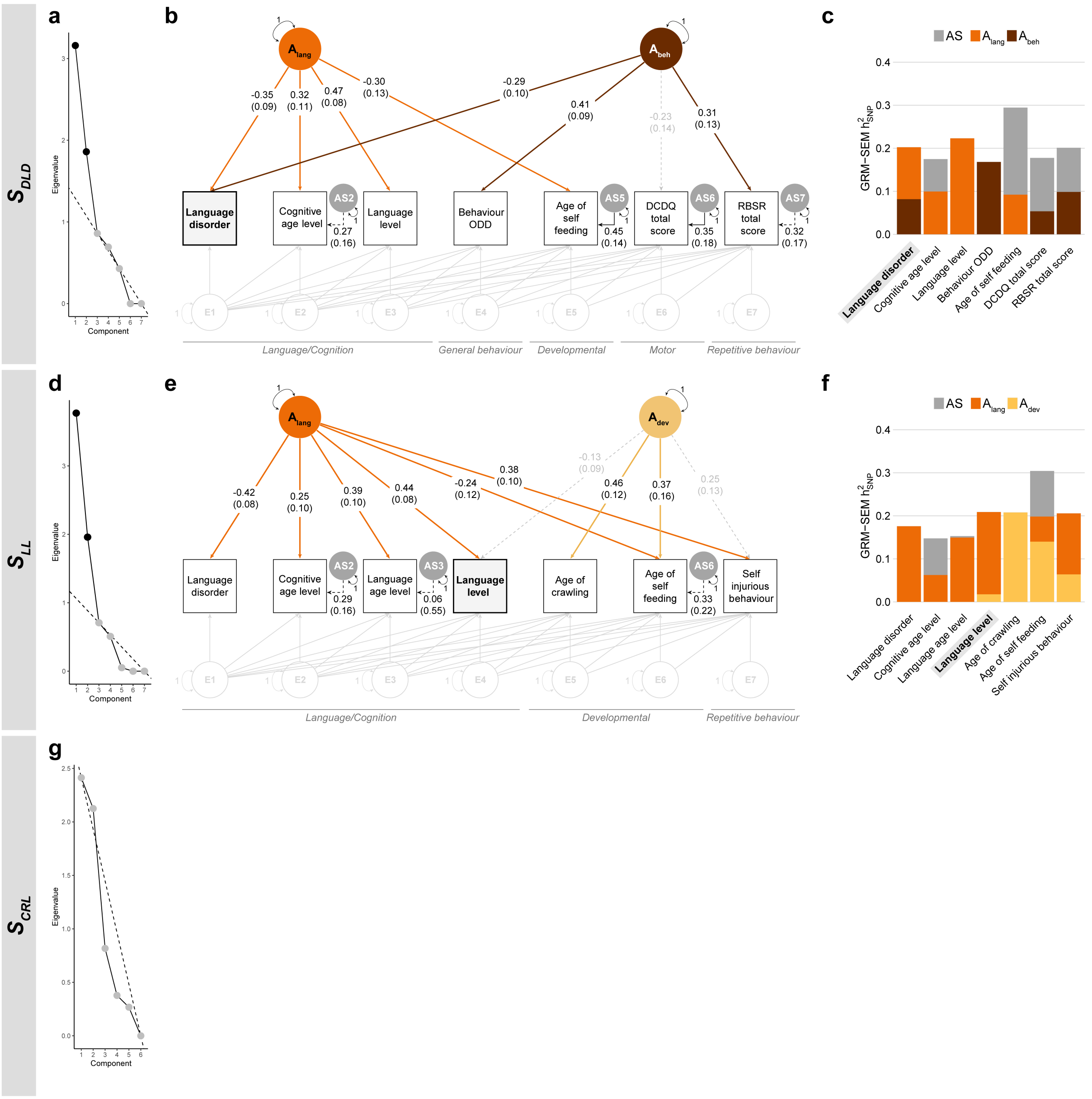

Supplementary Fig. 5 Multi-factor GRM-SEM models in SPARK. **a** Scree plot, **b** path diagram and **c** standardised genetic variance (GRM-SEM h^2_{SNP}) plot of the best-fitting GRM-SEM IPC model for the language disorder (S_{DLD}) subset. **d** Scree plot, **e** path diagram and **f** standardised genetic variance (GRM-SEM h^2_{SNP}) plot of the best-fitting GRM-SEM model for the language level (S_{LL}) subset. **g** Scree plot of the age of crawling (S_{CRL}) subset. **a,d,g** Scree plots are based on the eigenvalue decomposition of genetic correlations derived from a GRM-SEM Cholesky model, depicting the number of estimated shared genetic factors (in black) according to an optimal coordinate criterion. The dashed line indicates the "scree" of the plot (grey). **b,e** Observed measures are represented by squares and latent variables by circles (A: shared genetic factor, AS: specific genetic factor, E: residual factor). Single-headed arrows define factor loadings (shown with their corresponding SEs). The genetic part of the model has been modelled using an Independent Pathway model. Grey dotted and coloured solid arrows define shared genetic factor loadings with $p>0.05$ and $p\leq0.05$, respectively. Black dotted lines define specific genetic factor loadings with $p>0.05$. The residual part has been modelled using a Cholesky model (grey). **c,f** SEs for GRM-SEM h^2_{SNP} contributions have been omitted for clarity. Note that no GRM-SEM model was fitted to the third S_{CRL} (age of crawling) subset, as the number of genetic factors could not be unambiguously predicted by the optimal coordinate criterion. Abbreviations: A_{lang} (genetic language performance factor), A_{dev} (genetic developmental motor delay factor), A_{beh} (genetic behavioural-problems factor), DCDQ (Developmental Coordination Disorder Questionnaire), h^2_{SNP} (Single nucleotide polymorphism-based heritability), IPC (Independent Pathway-Cholesky GRM-SEM model), ODD (Oppositional Defiant Disorder), RBSR (Repetitive Behaviours Scale-Revised).

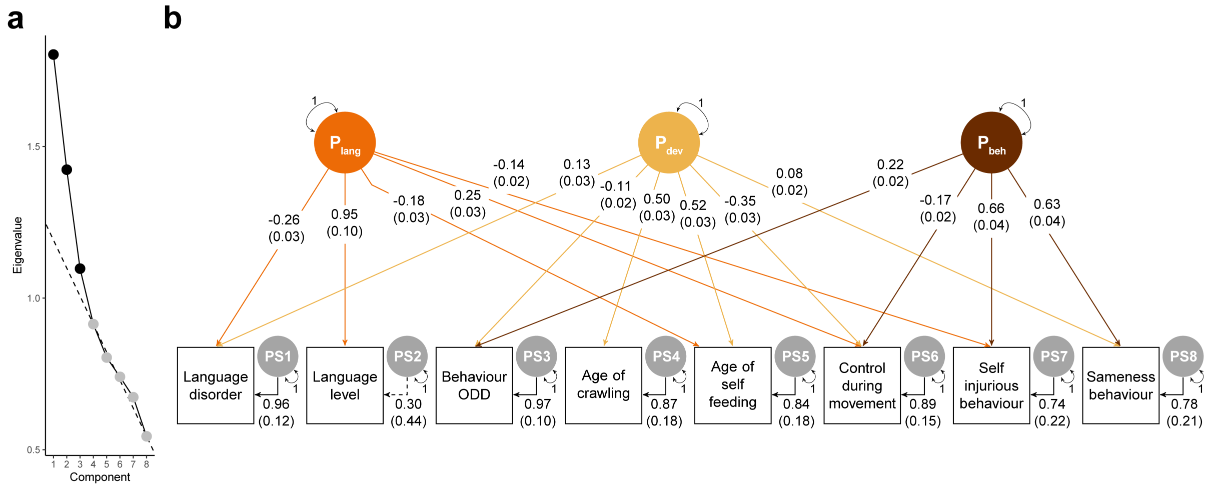

Supplementary Fig. 6 Phenotypic model in SPARK. **a** Scree plot of the eigenvalue decomposition of the phenotypic correlation matrix. **b** Path diagram depicting the phenotypic confirmatory factor analysis (CFA) model in SPARK. Observed measures are represented by squares and latent variables by circles (P: shared phenotypic factor, PS: specific phenotypic factor). For the shared phenotypic factors, solid single-headed arrows (factor loadings, shown with their corresponding SEs) define relationships between variables with p≤0.05. For the specific phenotypic factors, dotted and solid arrows define variances with p>0.05 and p≤0.05, respectively.

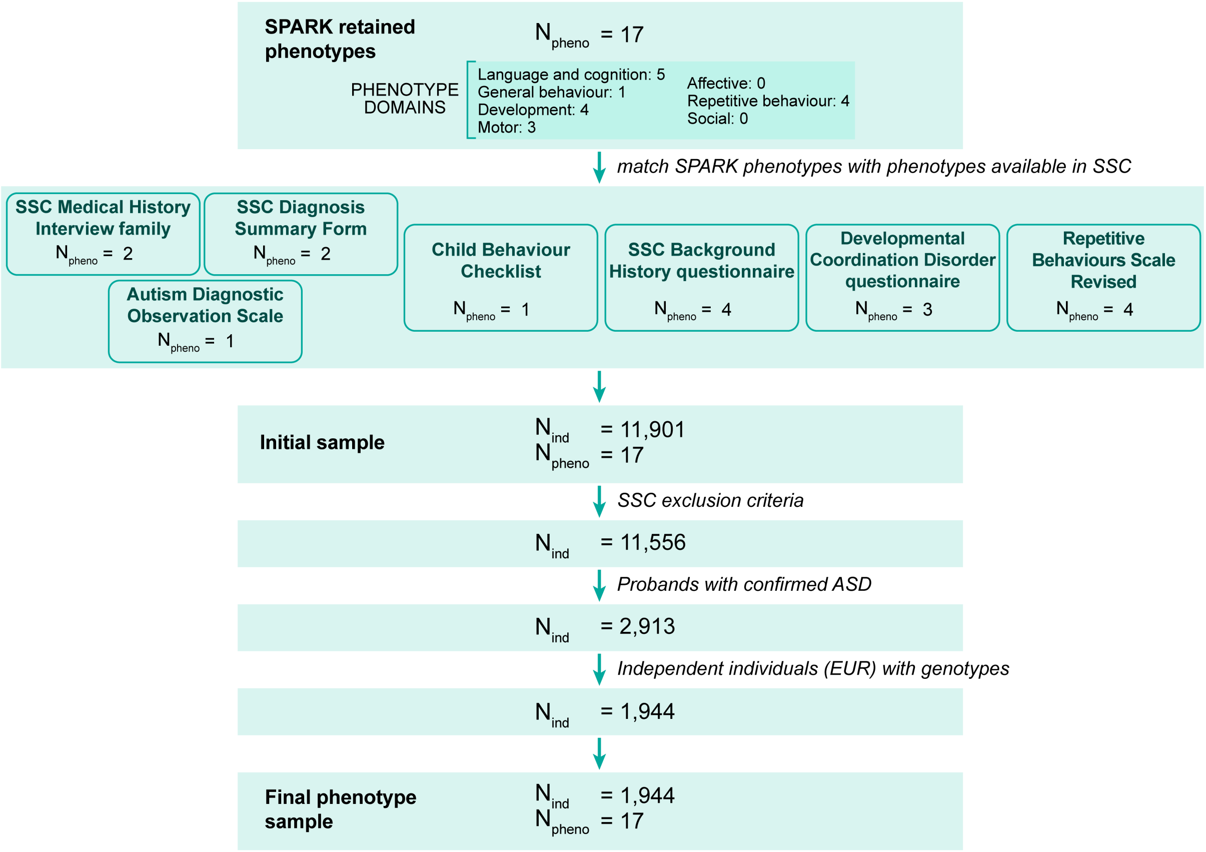

Supplementary Fig. 7 Individual and phenotype selection in the Simons Simplex Collection (SSC) sample. Individual and phenotype selection in the SSC sample. 17 phenotypes were selected, analogous to 17 phenotypes retained in SPARK (Fig. 2), from the SSC Medical History Interview (MEDHX fam, 2 language/cognition phenotypes), the SSC Diagnosis Summary Form (2 language/cognition phenotypes), Autism Diagnostic Interview-Revised (ADI-R, a language/cognition phenotype), the Child Behaviour Checklist (CBCL 6-18, a behavioural phenotype), the SSC Background History Questionnaire (BGHX, 4 developmental phenotypes), the Developmental Coordination Disorder Questionnaire (DCDQ, 3 motor phenotypes), and the Repetitive Behaviours Scale-Revised (RBSR, 4 repetitive behaviour phenotypes).

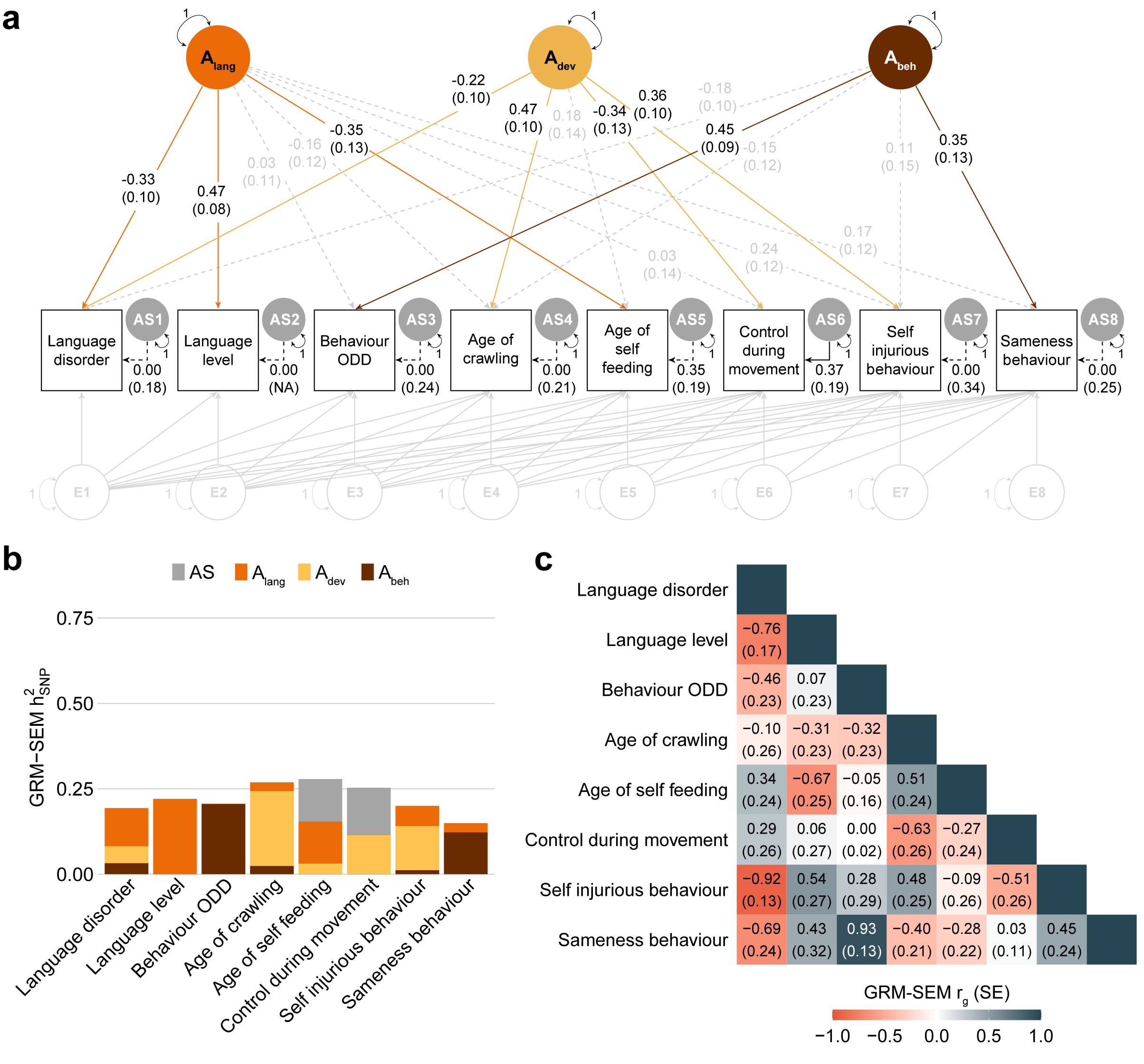

Supplementary Fig. 8 Bi-factor model for the best-fitting model in SPARK. **a** Path diagram depicting the bi-factor GRM-SEM model in the SPARK sample. Observed measures are represented by squares and latent variables by circles (A: shared genetic factor, AS: specific genetic factor, E: residual factor). Single-headed arrows define factor loadings (shown with their corresponding SEs). The genetic part of the model has been modelled using an Independent Pathway model (bi-factor model). Grey dotted and coloured solid arrows define shared genetic factor loadings with p>0.05 and p≤0.05, respectively. Black and solid dotted lines define specific genetic factor loadings with p>0.05 and p≤0.05, respectively. The residual part has been modelled using a Cholesky model (grey). **b** Corresponding standardised genetic variance (GRM-SEM h^2_{SNP}) plot. SEs for GRM-SEM h^2_{SNP} contributions have been omitted for clarity. **c** Corresponding correlogram of genetic correlations. Abbreviations: h^2_{SNP} (single nucleotide polymorphism-based heritability), IPC (Independent Pathway-Cholesky GRM-SEM model), ODD (Oppositional Defiant Disorder), r_g (genetic correlation).

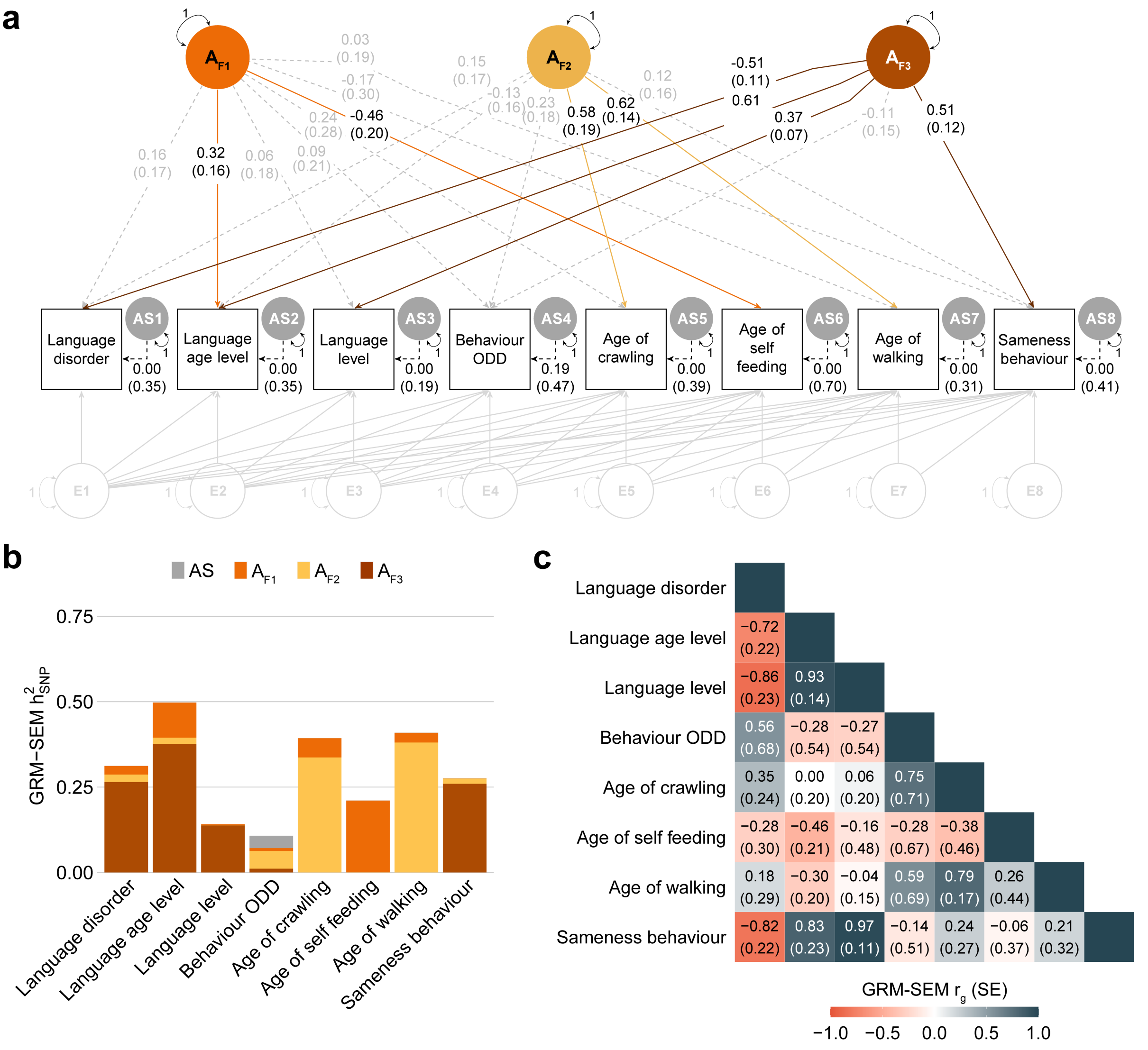

Supplementary Fig. 9 Bi-factor model for the best-fitting model in SSC. **a** Path diagram depicting the bi-factor GRM-SEM model in the SSC sample. Observed measures are represented by squares and latent variables by circles (A: shared genetic factor, AS: specific genetic factor, E: residual factor). Single-headed arrows define factor loadings (shown with their corresponding SEs). The genetic part of the model has been modelled using an Independent Pathway model (bi-factor model). Grey dotted and coloured solid arrows define shared genetic factor loadings with p>0.05 and p≤0.05, respectively. Black dotted lines define specific genetic factor loadings with p>0.05. The residual part has been modelled using a Cholesky model (grey). **b** Corresponding standardised genetic variance (GRM-SEM h^2_{SNP}) plot. SEs for GRM-SEM h^2_{SNP} contributions have been omitted for clarity. **c** Corresponding correlogram of genetic correlations. Abbreviations*:* h^2_{SNP} (Single nucleotide polymorphism-based heritability), IPC (Independent Pathway-Cholesky GRM-SEM model), r_g (genetic correlation).

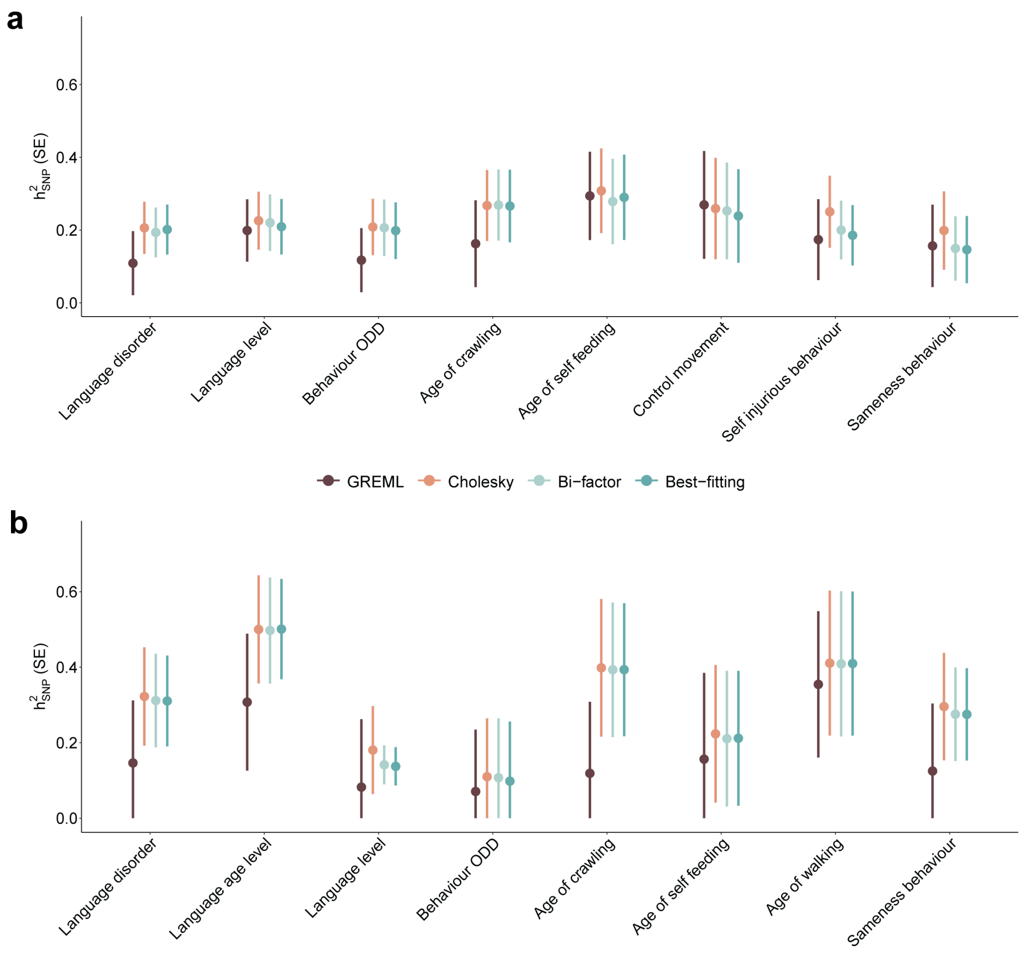

Supplementary Fig. 10 Comparison of heritability estimates between GREML and GRM-SEM. **a** h^2_{SNP} comparison across structural models fitted to the best-fitting model in SPARK. **b** h^2_{SNP} comparison across structural models fitted to the best-fitting model in SSC. **a,b** Comparison across univariate GREML (as implemented in GCTA) as well as GRM-SEM Cholesky, bi-factor and best-fitting multifactorial IPC models. Error bars represent standard errors. All analyses are based on transformed scores (categorical phenotypes: deviance residuals; continuous scores: rank-transformed residuals). Abbreviations: h^2_{SNP} (single nucleotide polymorphism-based heritability), IPC (Independent Pathway-Cholesky GRM-SEM model), ODD (Oppositional Defiant Disorder).

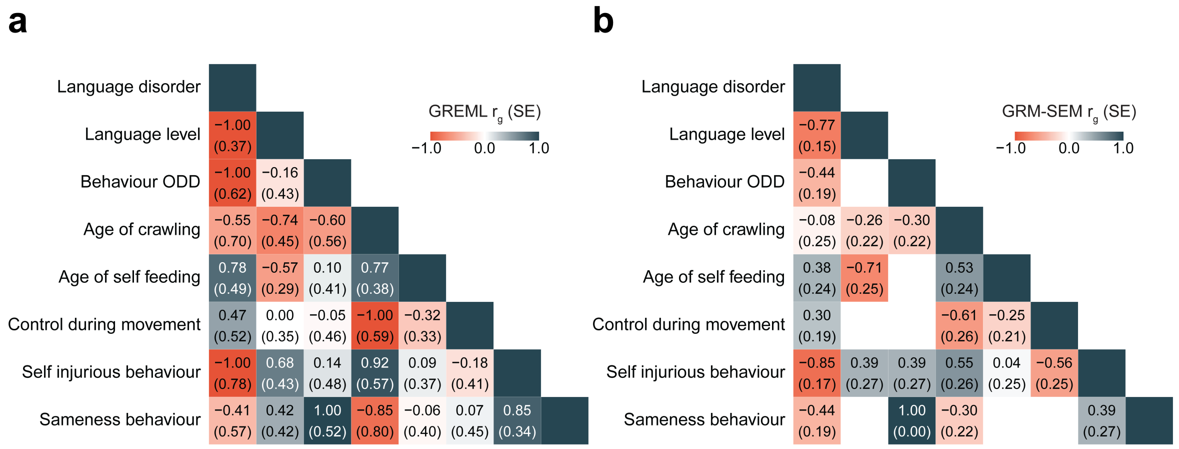

Supplementary Fig. 11 Comparison of genetic correlation estimates between GREML and GRM-SEM. **a** Genetic correlation estimates based on bivariate GREML (as implemented in GCTA) for the best-fitting IPC model in SPARK. **b** Genetic correlation estimates based on GRM-SEM for the best-fitting IPC model in SPARK. **a,b** All analyses are based on transformed scores (categorical phenotypes: deviance residuals; continuous scores: rank-transformed residuals). Abbreviations: r_g (genetic correlation), IPC (Independent Pathway-Cholesky GRM-SEM model), ODD (Oppositional Defiant Disorder).

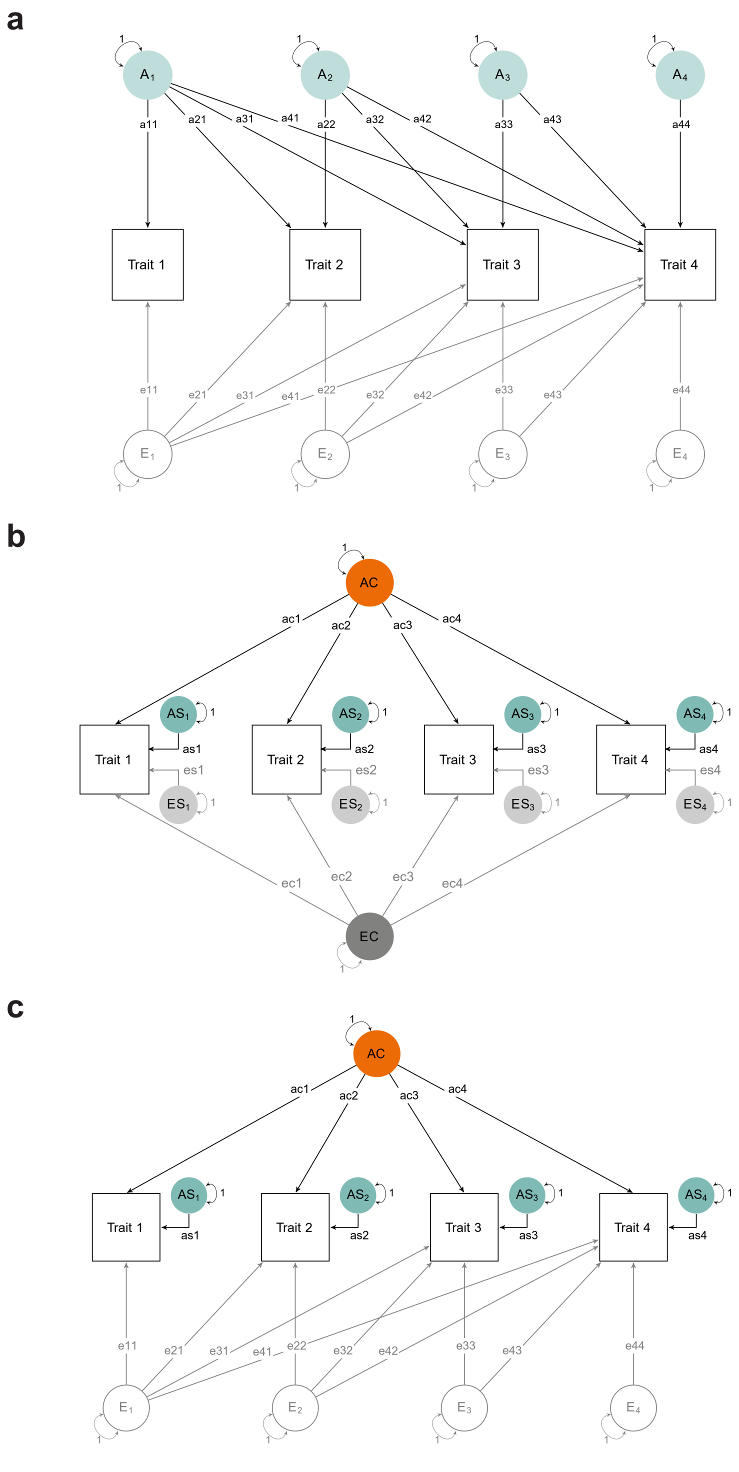

Supplementary Fig. 12 GRM-SEM multivariate model types**. a** Cholesky decomposition model (saturated). **b** Independent Pathway (IP) model. **c** Hybrid Independent Pathway-Cholesky (IPC) model, where the genetic variance is modelled with an IP structure and the residual variance with a Cholesky decomposition. **a-c** For all multivariate model types a hypothetical four-variate trait is illustrated. Observed measures are represented by squares and latent variables by circles (A: shared genetic factor, AS: specific genetic factor, E: residual factor). Single-headed arrows define factor loadings.

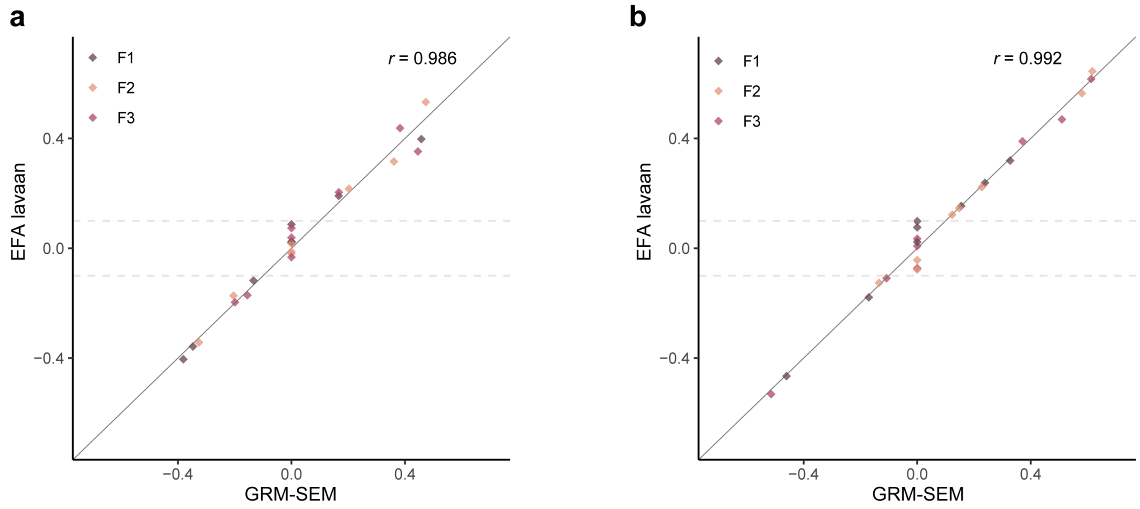

Supplementary Fig. 13 Comparison of factor loadings estimated with EFA lavaan and GRM-SEM. Factor loadings for genetic factors (F1-3) were compared between exploratory factor analysis (EFA) lavaan software (varimax rotation, DWLS algorithm) and GRM-SEM models fitted to the **a** best-fitting model in SPARK (Fig. 3) and **b** best-fitting model in SSC (Fig. 4). The correlation coefficient (r, Pearson) between EFA and GRM-SEM estimates is shown in the graph.

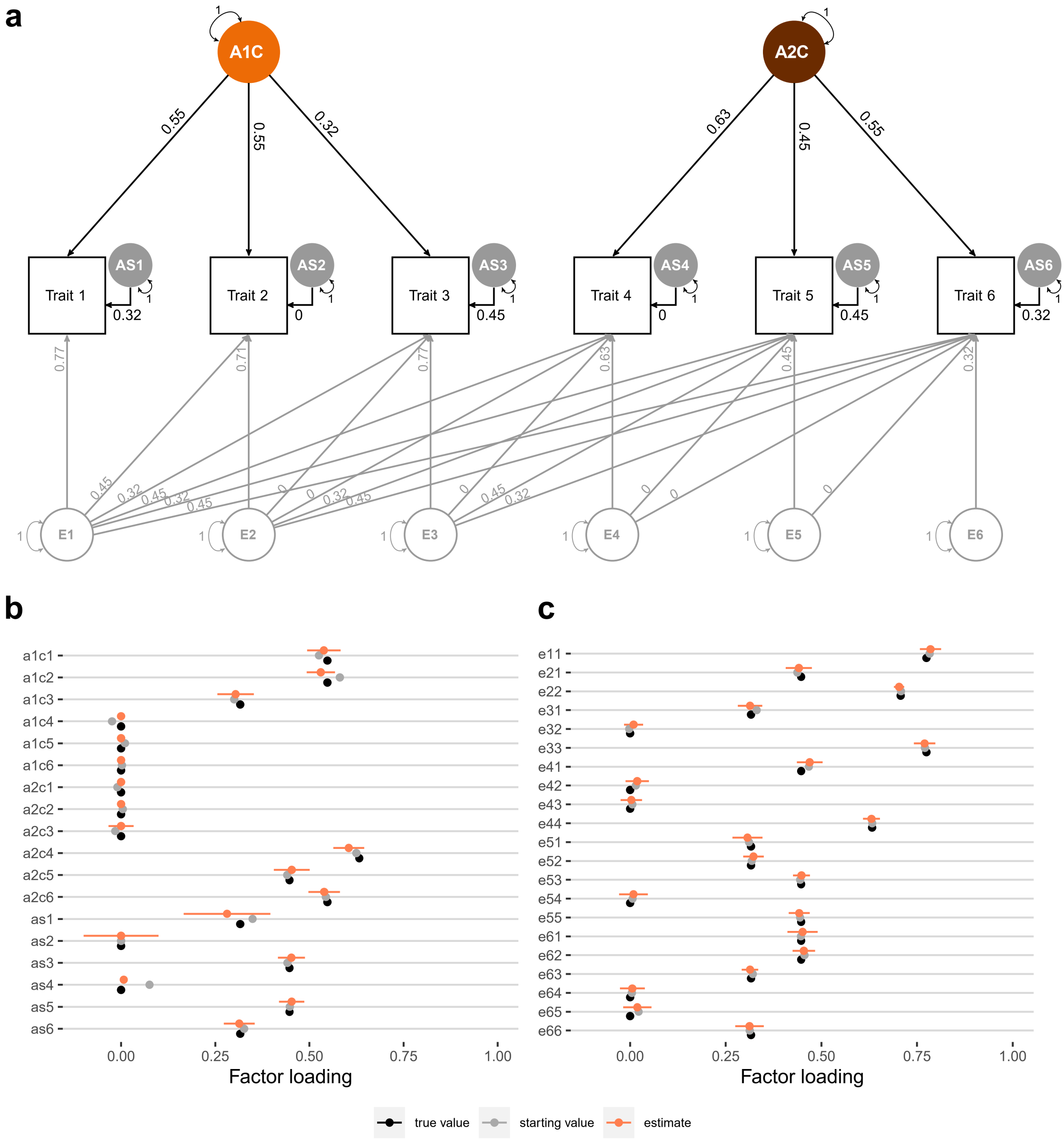

Supplementary Fig. 14 GRM-SEM simulations of a six-variate trait with two genetic factors without cross-loadings. **a** Path diagram of the simulated six-variate trait, assuming two genetic factors (A1C and A2C) without cross-loading, based on 2,000 individuals per trait and (for simplicity) 5,000 causal loci. **b** Genetic and **c** residual factor loadings with true values, median starting values and median estimated values (± Empirical SE) across 20 simulations based on a data-driven genomic covariance modelling approach (Fig. 1b).

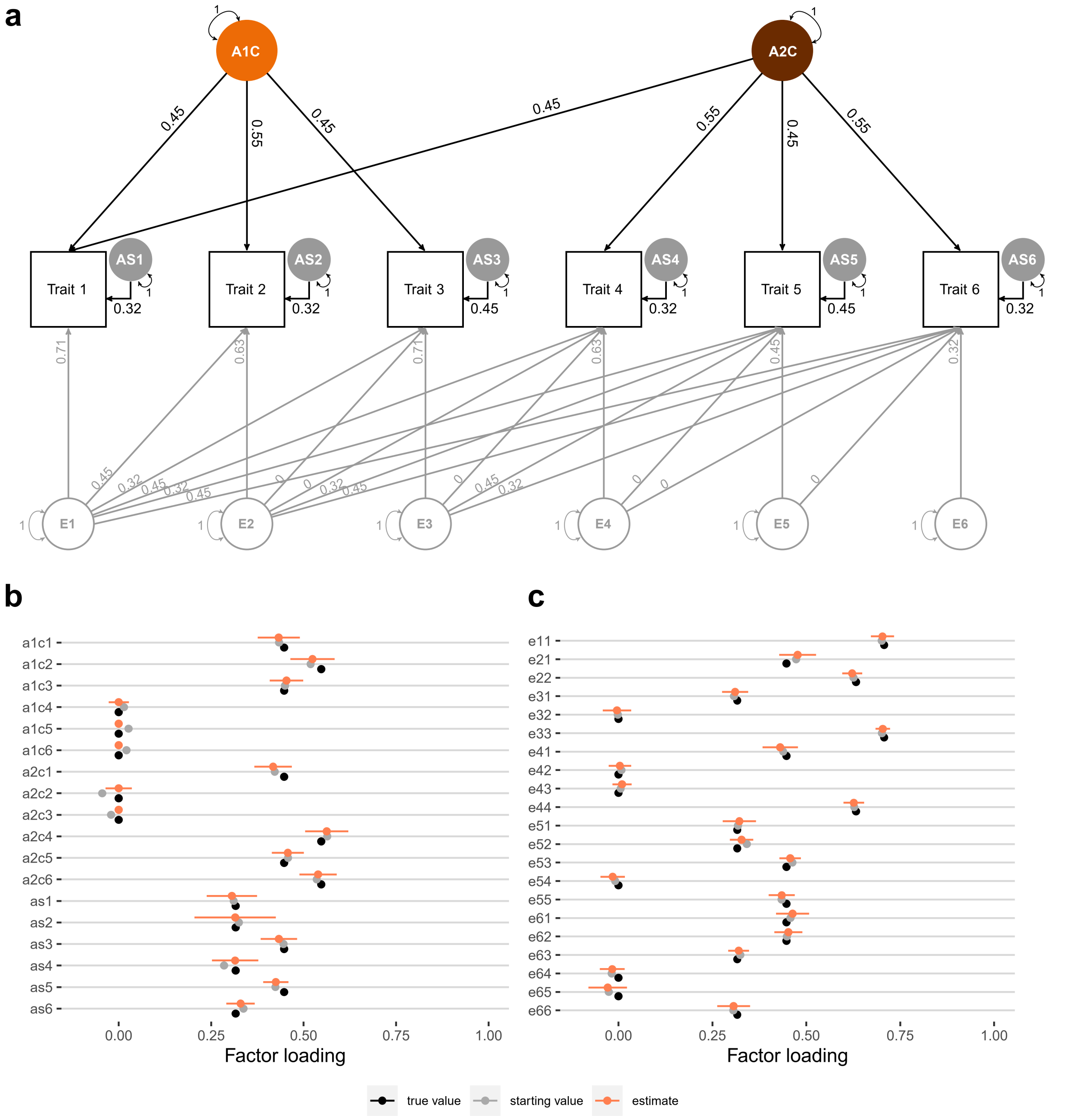

Supplementary Fig. 15 GRM-SEM simulations of a six-variate trait with two genetic factors with cross-loading. **a** Path diagram of a six-variate trait, assuming two genetic factors (A1C and A2C) with cross-loading, based on 2,000 individuals per trait and (for simplicity) 5,000 causal loci. **b** Genetic and **c** residual factor loadings with true values, median starting values and median estimated values (± Empirical SE) across 20 simulations based on a data-driven genomic covariance modelling approach (Fig. 1b).

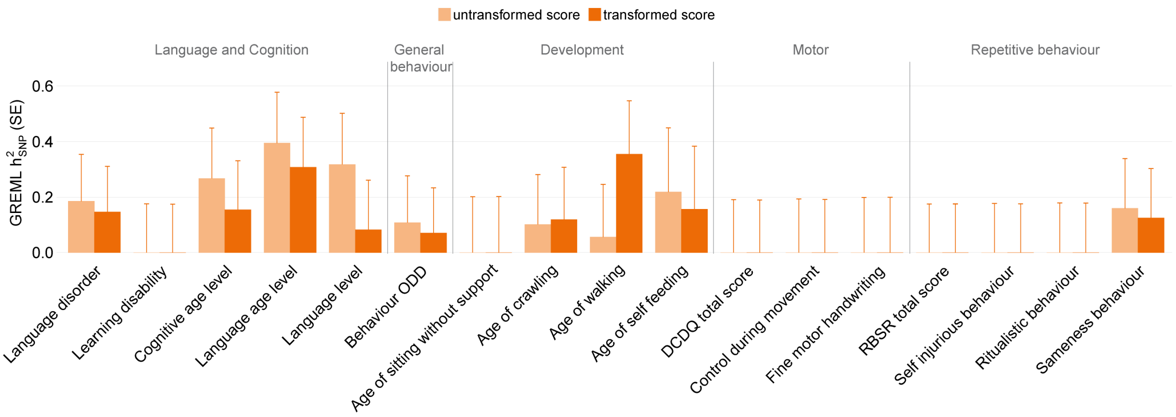

Supplementary Fig. 16 GREML heritability (h^2_{SNP}) estimates of phenotypes in SSC. The error bars represent standard errors. GREML h^2_{SNP} estimates for categorical phenotypes are shown for untransformed scores (light orange) and deviance residuals (dark orange). GREML h^2_{SNP} estimates for continuous phenotypes are shown for untransformed scores (light orange) and rank-transformed residuals (dark orange). GREML h^2_{SNP} estimates were adjusted for sex, age, age squared, and ten ancestry-informative principal components. For a detailed description of SSC phenotypes see Supplementary Data 6. Abbreviations: DCDQ (Developmental Coordination Disorder Questionnaire), h^2_{SNP} (single nucleotide polymorphism-based heritability), ODD (oppositional defiant disorder), RBSR (Repetitive Behaviour Scale-Revised).

untransformed scores
transformed scores

Spearman ρ
−1.0 0.0 1.0

		Language disorder	Learning disability	Cognitive age level	Language age level	Language level	Behaviour ODD	Age of sitting wo support	Age of crawling	Age of walking	Age of self feeding	DCDQ total score	Control during movement	Fine motor handwriting	RBSR total score	Self injurious behaviour	Ritualistic behaviour	Sameness behaviour
Language and Cognition	Language disorder	0.99	0.06	−0.06	−0.29	−0.26	−0.21	0.06	0.06	0.07	0.06	−0.13	−0.11	−0.16	0.04	0.03	−0.01	0.01
	Learning disability	0.24	0.98	−0.35	−0.27	−0.07	0.35	0.02	0.04	0.08	0.11	−0.10	−0.07	−0.18	0.10	0.06	0.06	0.07
	Cognitive age level	−0.22	−0.25	0.99	0.44	0.24	−0.22	−0.09	−0.09	−0.10	−0.19	0.26	0.22	0.30	−0.16	−0.11	−0.06	−0.11
	Language age level	−0.45	−0.11	0.40	0.96	0.54	−0.18	−0.04	−0.03	−0.06	−0.18	0.21	0.18	0.30	−0.09	−0.07	0.02	−0.03
	Language level	−0.32	0.00	0.31	0.67	0.83	0.01	−0.03	−0.01	−0.03	−0.15	0.26	0.21	0.33	−0.07	−0.12	0.11	0.00
General behaviour	Behaviour ODD	−0.07	0.06	0.03	0.14	0.13	0.98	−0.05	−0.05	−0.02	0.05	0.01	0.04	−0.03	0.07	0.09	0.03	0.05
Developmental	Age of sitting wo support	0.04	0.04	−0.12	−0.04	−0.02	−0.03	0.98	0.60	0.48	0.25	−0.20	−0.18	−0.16	0.06	0.05	0.03	0.06
	Age of crawling	0.05	0.07	−0.12	−0.03	0.00	−0.03	0.61	0.99	0.58	0.27	−0.18	−0.18	−0.12	0.01	0.01	0.00	0.01
	Age of walking	0.07	0.09	−0.13	−0.06	−0.04	−0.04	0.48	0.58	0.99	0.27	−0.21	−0.21	−0.15	0.02	0.05	−0.01	0.00
	Age of self feeding	0.13	0.03	−0.14	−0.17	−0.21	−0.07	0.25	0.27	0.27	1.00	−0.22	−0.21	−0.18	0.09	0.07	0.05	0.05
Motor	DCDQ total score	−0.14	−0.13	0.33	0.23	0.28	0.01	−0.20	−0.18	−0.21	−0.22	1.00	0.90	0.78	−0.30	−0.21	−0.17	−0.21
	Control during movement	−0.11	−0.09	0.29	0.19	0.24	0.05	−0.18	−0.18	−0.22	−0.22	0.89	1.00	0.53	−0.23	−0.14	−0.15	−0.17
	Fine motor handwriting	−0.19	−0.18	0.37	0.33	0.38	−0.01	−0.14	−0.11	−0.14	−0.19	0.78	0.52	0.99	−0.27	−0.21	−0.12	−0.18
Repetitive behaviour	RBSR total score	0.05	0.11	−0.14	−0.10	−0.13	0.12	0.06	0.01	0.01	0.09	−0.30	−0.23	−0.27	0.98	0.59	0.80	0.87
	Self injurious behaviour	0.03	0.09	−0.13	−0.07	−0.13	0.15	0.05	0.01	0.05	0.07	−0.22	−0.15	−0.22	0.60	0.99	0.32	0.40
	Ritualistic behaviour	0.00	0.07	−0.02	0.01	0.05	0.08	0.03	0.00	−0.01	0.06	−0.18	−0.15	−0.13	0.80	0.33	0.99	0.71
	Sameness behaviour	0.00	0.11	−0.11	−0.02	−0.01	0.12	0.07	0.01	0.00	0.05	−0.22	−0.17	−0.18	0.87	0.41	0.72	0.99

Supplementary Fig. 17 Phenotypic correlations of phenotypes in SPARK. Phenotypic correlations (Spearman's rho) across measures with h^2_{SNP} (p≤0.1), including transformed scores (lower triangle) and untransformed scores (upper triangle). The diagonal shows the correlation between the untransformed and transformed scores. Transformed scores for categorical phenotypes are based on deviance residuals and transformed scores for continuous phenotypes on rank-transformed residuals. Transformed scores were adjusted for sex, age, age squared, and ten ancestry-informative principal components. Abbreviations: DCDQ (Developmental Coordination Disorder Questionnaire), h^2_{SNP} (single nucleotide polymorphism-based heritability), ODD (oppositional defiant disorder), RBSR (Repetitive Behaviour Scale-Revised), wo (without).

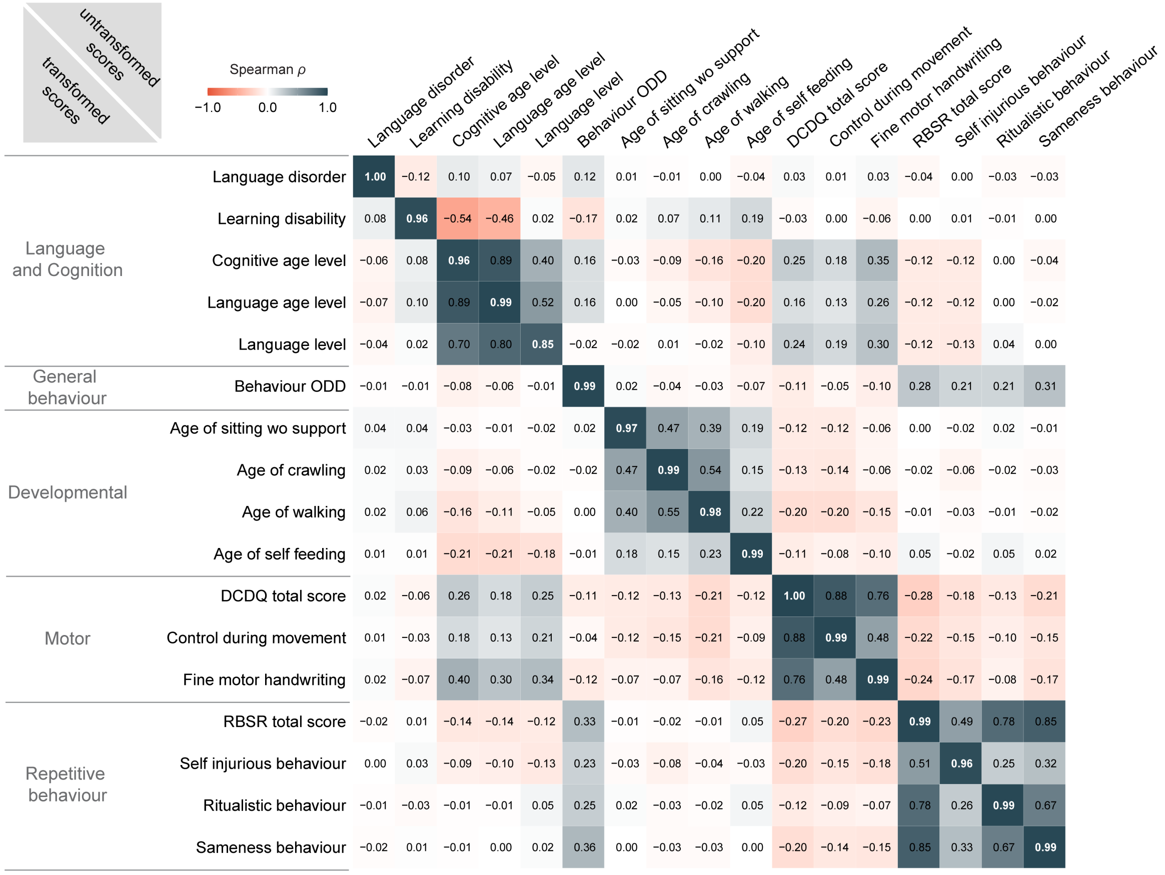

Supplementary Fig. 18 Phenotypic correlations of phenotypes in SSC. Phenotypic correlations (Spearman's rho) of SSC measures matched to SPARK), including transformed scores (lower triangle) and untransformed scores (upper triangle). The diagonal shows the correlation between the untransformed and transformed scores. Transformed scores for categorical phenotypes are based on deviance residuals and transformed scores for continuous phenotypes on rank-transformed residuals. Transformed scores were adjusted for sex, age, age squared, and ten ancestry-informative principal components. Abbreviations: DCDQ (Developmental Coordination Disorder Questionnaire), h^2_{SNP} (single nucleotide polymorphism-based heritability), ODD (oppositional defiant disorder), RBSR (Repetitive Behaviour Scale-Revised), wo (without).

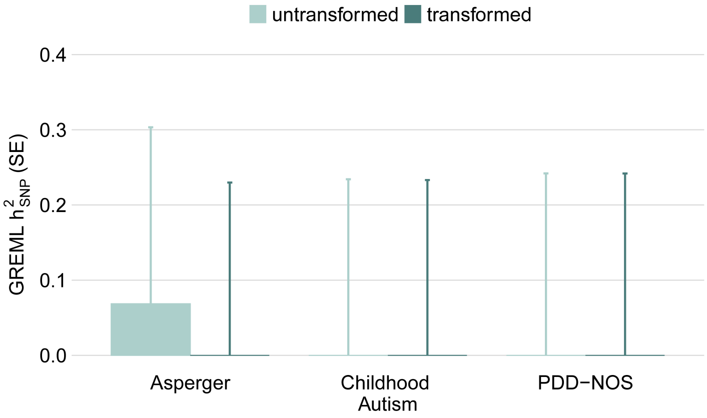

Supplementary Fig. 19 GREML heritability (h^2_{SNP}) estimates of dichotomised ASD subcategories in SPARK. The error bars represent standard errors. GREML h^2_{SNP} estimates for transformed scores, adjusted for sex, age, age squared, and ten ancestry-informative principal components. From a total sample of 5,331 ASD-unrelated individuals with phenotypic and genotyping information, 716 individuals had information on Asperger (566 males, 150 females), 624 individuals had information on Childhood Autism (509 males, 115 females) and 414 individuals had information on PDD-NOS (334 males, 80 females). Contrasts were coded as follows: Asperger (Asperger=1, Childhood Autism=0, PDD-NOS=0, not-available=NA), Childhood Autism (Asperger=0, Childhood Autism=1, PDD-NOS=0, not-available=NA) and PDD-NOS (Asperger=0, Childhood Autism=0, PDD-NOS=1, not-available=NA). Abbreviations: h^2_{SNP} (single nucleotide polymorphism-based heritability), PDD-NOS (Pervasive Developmental Disorder Not Otherwise Specified).

Supplementary References

1. Morris, T. P., White, I. R. & Crowther, M. J. Using simulation studies to evaluate statistical methods. *Statistics in Medicine* **38**, 2074–2102 (2019).
2. Feliciano, P. *et al.* SPARK: A US Cohort of 50,000 Families to Accelerate Autism Research. *Neuron* **97**, 488–493 (2018).
3. Ge, T., Chen, C.-Y., Ni, Y., Feng, Y.-C. A. & Smoller, J. W. Polygenic prediction via Bayesian regression and continuous shrinkage priors. *Nat Commun* **10**, 1776 (2019).
4. Lee, J. J. *et al.* Gene discovery and polygenic prediction from a genome-wide association study of educational attainment in 1.1 million individuals. *Nat Genet* **50**, 1112–1121 (2018).
5. Chang, C. C. *et al.* Second-generation PLINK: rising to the challenge of larger and richer datasets. *GigaScience* **4**, s13742-015-0047–8 (2015).
