# Supplementary material for: Structural models of genome-wide covariance identify multiple common dimensions in autism
Source: Nat Commun. 2024 Feb 27;15:1770. doi: 10.1038/s41467-024-46128-8 (PMC10899248; doi:10.1038/s41467-024-46128-8)
Supplement: Supplementary file 3 — Description of Additional Supplementary Files [file 41467_2024_46128_MOESM3_ESM.pdf]

Description of Additional Supplementary Files

Supplementary Data 1: Variable description and SNP heritability of phenotypes in the Simons Foundation Powering Autism Research for Knowledge (SPARK) sample

Supplementary Data 2: Identification of phenotype subsets in SPARK

Supplementary Data 3: Model fit comparison in exploratory models in SPARK

Supplementary Data 4: Model fit comparison

Supplementary Data 5: Best-fitting structural model in SPARK

Supplementary Data 6: Variable description and SNP heritability of phenotypes in the Simons Simplex Collection (SSC) sample

Supplementary Data 7: Best-fitting structural model in SSC

Supplementary Data 8: Comparison of genetic EFA factor loadings

Supplementary Data 9: Comparison of genetic factor loadings across EFA and GRM-SEM

Supplementary Data 10: GRM-SEM simulations for a six-variate trait with two genetic factors without cross-loadings (2,000 individuals per phenotype, 5,000 causal loci): Factor loadings

Supplementary Data 11: GRM-SEM simulations for a six-variate trait with two genetic factors without cross-loadings (2,000 individuals per phenotype, 5,000 causal loci): Genetic and residual variance

Supplementary Data 12: GRM-SEM simulations for a six-variate trait with two common genetic factors with cross-loading (2,000 individuals per phenotype, 5,000 causal loci): Factor loadings

Supplementary Data 13: GRM-SEM simulations for a six-variate trait with two genetic factors with cross-loading (2,000 individuals per phenotype, 5,000 causal loci): Genetic and residual variance
