# Supplementary material for: Structural models of genome-wide covariance identify multiple common dimensions in autism
Source: Nat Commun. 2024 Feb 27;15:1770. doi: 10.1038/s41467-024-46128-8 (PMC10899248; doi:10.1038/s41467-024-46128-8)
Supplement: Supplementary file 5 — Reporting Summary [file 41467_2024_46128_MOESM5_ESM.pdf]

nature portfolio

Corresponding author(s): Beate St Pourcain
Last updated by author(s): Dec 19, 2023

Reporting Summary

Nature Portfolio wishes to improve the reproducibility of the work that we publish. This form provides structure for consistency and transparency in reporting. For further information on Nature Portfolio policies, see our Editorial Policies and the Editorial Policy Checklist.

Statistics

For all statistical analyses, confirm that the following items are present in the figure legend, table legend, main text, or Methods section.

n/a	Confirmed	
☐	☒	The exact sample size (*n*) for each experimental group/condition, given as a discrete number and unit of measurement
☐	☒	A statement on whether measurements were taken from distinct samples or whether the same sample was measured repeatedly
☐	☒	The statistical test(s) used AND whether they are one- or two-sided<br>*Only common tests should be described solely by name; describe more complex techniques in the Methods section.*
☐	☒	A description of all covariates tested
☐	☒	A description of any assumptions or corrections, such as tests of normality and adjustment for multiple comparisons
☐	☒	A full description of the statistical parameters including central tendency (e.g. means) or other basic estimates (e.g. regression coefficient) AND variation (e.g. standard deviation) or associated estimates of uncertainty (e.g. confidence intervals)
☐	☒	For null hypothesis testing, the test statistic (e.g. *F*, *t*, *r*) with confidence intervals, effect sizes, degrees of freedom and *P* value noted<br>*Give P values as exact values whenever suitable.*
☐	☒	For Bayesian analysis, information on the choice of priors and Markov chain Monte Carlo settings
☒	☐	For hierarchical and complex designs, identification of the appropriate level for tests and full reporting of outcomes
☐	☒	Estimates of effect sizes (e.g. Cohen's *d*, Pearson's *r*), indicating how they were calculated

Our web collection on statistics for biologists contains articles on many of the points above.

Software and code

Policy information about availability of computer code

Data collection

No software has been used for data collection

Data analysis

Software for data analysis
- Plink software for genotyping quality control of SPARK and SSC: PLINK 1.9 (cog-genomics.org)
- PRScs analysis: getian107/PRScs (github.com)
- GCTA-GREML: GCTA v 1.93 (cnsgenomics.com)
R packages for data analysis
(1) R:stats package, version 4.0.2 CRAN
(2) R:base package, version 4.0.2 CRAN
(3) R:nFactors package, version 2.4.1 CRAN
(4) R:psych package, version 2.2.3 CRAN
(4) R:lavaan package, version 0.6-10 CRAN
(5) R:grmsem package, version 1.1.2 gitlab: https://gitlab.gwdg.de/beate.stpourcain/grmsem

Code used for the analysis conducted in this study is available in GitLab: https://gitlab.gwdg.de/pghc/disentangling-asd-heterogeneity-using-grm-sem/nature-communications-2023

For manuscripts utilizing custom algorithms or software that are central to the research but not yet described in published literature, software must be made available to editors and reviewers. We strongly encourage code deposition in a community repository (e.g. GitHub). See the Nature Portfolio guidelines for submitting code & software for further information.

Data

Policy information about availability of data

All manuscripts must include a data availability statement. This statement should provide the following information, where applicable:

- Accession codes, unique identifiers, or web links for publicly available datasets
- A description of any restrictions on data availability
- For clinical datasets or third party data, please ensure that the statement adheres to our policy

Genotype and phenotype data from the SPARK and SSC cohorts are available upon application and approval from the Simons Foundation Autism Research Initiative (SFARI) (https://www.sfari.org/resource/autism-cohorts/). Approved researchers can obtain the SPARK and SSC population dataset described in this study by applying at https://base.sfari.org. Detailed reasons for controlled access and details of any restrictions imposed on data use via data use agreements have been outlined in the RESEARCHER DISTRIBUTION AGREEMENT of the Simons Collection (https://s3.amazonaws.com/sf-web-assets-prod/wp-content/uploads/sites/2/2021/06/15165956/SFARI_RDA.pdf) to ensure compliance with data-protection. The timeframe for response of the SFARI Collection to data requests is rapid (usually < 2 months).

GWAS summary statistics for educational attainment (EA3, Lee et al. 2018) were accessed through the Social Science Genetic Association Consortium (SSGAC, https://thessgac.com/papers/3).

Research involving human participants, their data, or biological material

Policy information about studies with human participants or human data. See also policy information about sex, gender (identity/presentation), and sexual orientation and race, ethnicity and racism.

Reporting on sex and gender	Sex was based on self-reported and genotypically assigned information. Individuals with sex mismatch from these two sources were excluded.
Reporting on race, ethnicity, or other socially relevant groupings	Ancestry was based on genotypically assigned information. Individuals with a European genetically-informed ancestry were included in this study.
Population characteristics	SPARK: 5,331 unrelated individuals (79.85% males, median age: 9 years) of European ancestry diagnosed with ASD, with genetic and phenotype information were included in the study. A genetic-relationship-matrix based on directly genotyped markers (N_SNPs=450,491) was created in PLINK, applying a relationship cut-off of 0.05.<br>SSC: 1,946 unrelated individuals (86.33% males, median age: 9 years) of European ancestry diagnosed with ASD with genetic and ASD phenotype information were included in the study. A genetic-relationship-matrix based on directly genotyped markers (N_SNPs=457,961) was created in PLINK, applying a relationship cut-off of 0.05.
Recruitment	This study used previously collected datasets and did not prospectively recruit subjects.
Ethics oversight	We received ethical approval to access and analyse pre-collected de-identified genotype and phenotype data from SPARK and SSC cohorts from the Radboud University Ethics Committee Social Science. All analyses were restricted to individuals with ASD with phenotypic and genetic information.

Note that full information on the approval of the study protocol must also be provided in the manuscript.

Field-specific reporting

Please select the one below that is the best fit for your research. If you are not sure, read the appropriate sections before making your selection.

☒ Life sciences ☐ Behavioural & social sciences ☐ Ecological, evolutionary & environmental sciences

For a reference copy of the document with all sections, see nature.com/documents/nr-reporting-summary-flat.pdf

Life sciences study design

All studies must disclose on these points even when the disclosure is negative.

Sample size	From the SPARK cohort, we included 5,331 unrelated individuals with an autism diagnosis. From the SSC cohort, we included 1,946 unrelated individuals with an autism diagnosis. Sample size for both cohorts was determined using the number of individuals with genetically-informed European ancestry that had genetic and phenotypic data after quality control described in Supplementary Methods 1 and 2.
Data exclusions	We excluded individuals who did not pass genetic quality control, as described in Supplementary Methods 1 for SPARK and Supplementary Methods 2 for SSC. We further described the selection of individuals according to SSC and SPARK exclusion criteria.
Replication	One replication attempt was carried out, following up the SPARK results in the SSC sample. GREML and GRM-SEM findings in SPARK (GRM-SEM identifies multivariate models capturing multiple phenotypes and associations) were largely replicated in the SSC sample. Similarities and differences in models across samples are discussed in the Discussion section of the manuscript.

Randomization

No randomisation was required for GREML or GRM-SEM analyses. This study adopted a case-only design, analysing symptom variation across individuals with ASD for quantitative and qualitative scores. All scores were adjusted for sex, age, age squared, and ten ancestry-informative principal components. ASD cases were diagnosed using DSM criteria and standard diagnostic instruments.

Blinding

No blinding was conducted in this study as we adopted a cohort-design rather than an experimental design.

Reporting for specific materials, systems and methods

We require information from authors about some types of materials, experimental systems and methods used in many studies. Here, indicate whether each material, system or method listed is relevant to your study. If you are not sure if a list item applies to your research, read the appropriate section before selecting a response.

Materials & experimental systems

n/a	Involved in the study
☒	☐ Antibodies
☒	☐ Eukaryotic cell lines
☒	☐ Palaeontology and archaeology
☒	☐ Animals and other organisms
☒	☐ Clinical data
☒	☐ Dual use research of concern
☒	☐ Plants

Methods

n/a	Involved in the study
☒	☐ ChIP-seq
☒	☐ Flow cytometry
☒	☐ MRI-based neuroimaging

Plants

Seed stocks

Report on the source of all seed stocks or other plant material used. If applicable, state the seed stock centre and catalogue number. If plant specimens were collected from the field, describe the collection location, date and sampling procedures.

Novel plant genotypes

Describe the methods by which all novel plant genotypes were produced. This includes those generated by transgenic approaches, gene editing, chemical/radiation-based mutagenesis and hybridization. For transgenic lines, describe the transformation method, the number of independent lines analyzed and the generation upon which experiments were performed. For gene-edited lines, describe the editor used, the endogenous sequence targeted for editing, the targeting guide RNA sequence (if applicable) and how the editor was applied.

Authentication

Describe any authentication procedures for each seed stock used or novel genotype generated. Describe any experiments used to assess the effect of a mutation and, where applicable, how potential secondary effects (e.g. second site T-DNA insertions, mosiacism, off-target gene editing) were examined.
